# Effect of genetic variants and traits related to glucose metabolism and their interaction with obesity on breast and colorectal cancer risk among postmenopausal women

**DOI:** 10.1186/s12885-017-3284-7

**Published:** 2017-04-26

**Authors:** Su Yon Jung, Eric M. Sobel, Jeanette C. Papp, Zuo-Feng Zhang

**Affiliations:** 1 Translational Sciences Section, Jonsson Comprehensive Cancer Center, School of Nursing, University of California Los Angeles, 700 Tiverton Ave, 3-264 Factor Building, Los Angeles, CA 90095 USA; 2 Department of Human Genetics, David Geffen School of Medicine, University of California Los Angeles, Los Angeles, CA USA; 3 Department of Epidemiology, Fielding School of Public Health, University of California Los Angeles, Los Angeles, CA USA

**Keywords:** Glucose metabolism–related genetic variant, Obesity, Physical activity, High-fat diet, Breast cancer, Colorectal cancer, Postmenopausal women

## Abstract

**Background:**

Impaired glucose metabolism–related genetic variants and traits likely interact with obesity and related lifestyle factors, influencing postmenopausal breast and colorectal cancer (CRC), but their interconnected pathways are not fully understood. By stratifying via obesity and lifestyles, we partitioned the total effect of glucose metabolism genetic variants on cancer risk into two putative mechanisms: 1) indirect (risk-associated glucose metabolism genetic variants mediated by glucose metabolism traits) and 2) direct (risk-associated glucose metabolism genetic variants through pathways other than glucose metabolism traits) effects.

**Method:**

Using 16 single-nucleotide polymorphisms (SNPs) associated with glucose metabolism and data from 5379 postmenopausal women in the Women’s Health Initiative Harmonized and Imputed Genome-Wide Association Studies, we retrospectively assessed the indirect and direct effects of glucose metabolism-traits (fasting glucose, insulin, and homeostatic model assessment–insulin resistance [HOMA-IR]) using two quantitative tests.

**Results:**

Several SNPs were associated with breast cancer and CRC risk, and these SNP–cancer associations differed between non-obese and obese women. In both strata, the direct effect of cancer risk associated with the SNP accounted for the majority of the total effect for most SNPs, with roughly 10% of cancer risk due to the SNP that was from an indirect effect mediated by glucose metabolism traits. No apparent differences in the indirect (glucose metabolism-mediated) effects were seen between non-obese and obese women. It is notable that among obese women, 50% of cancer risk was mediated via glucose metabolism trait, owing to two SNPs: in breast cancer, in relation to *GCKR* through glucose, and in CRC, in relation to *DGKB/TMEM195* through HOMA-IR.

**Conclusions:**

Our findings suggest that glucose metabolism genetic variants interact with obesity, resulting in altered cancer risk through pathways other than those mediated by glucose metabolism traits.

**Electronic supplementary material:**

The online version of this article (doi:10.1186/s12885-017-3284-7) contains supplementary material, which is available to authorized users.

## Background

Breast cancer is the most commonly occurring cancer and the second most common cause of cancer-related deaths in the United States [1]. Colorectal cancer (CRC) is the second most commonly diagnosed cancer and one of the leading causes of cancer-related mortality throughout the world [2]. Impaired glucose metabolism, i.e. insulin resistance (IR), is characterized by hyperinsulinemia and hyperglycemia, and demonstrates strong associations with breast cancer and CRC [3–8]. The association is particularly strong in postmenopausal women, in whom high insulin levels have been associated with a twofold increase in breast cancer risk [9, 10]. The homeostatic model assessment–insulin resistance (HOMA-IR) reflecting high blood levels of insulin and glucose is positively associated with breast cancer in the postmenopausal women [11].

Besides its importance in glucose homeostasis, insulin is an essential hormone in anabolic processes in early cell growth and development, directly through the insulin receptor and indirectly through the insulin-like growth factor receptor [12, 13]. Insulin receptors that are mainly found in adipose tissues, muscle, and liver cells are overexpressed in breast cancer and CRC cells. This overexpression results in the enhanced anabolic state necessary for cell proliferation, differentiation, and anti-apoptosis, via abnormal stimulation of multiple signaling pathways, including the phosphatidylinositol 3-kinase (PI3K)/serine/threonine-specific protein kinase (Akt) and mitogen-activated protein kinase (MAPK) pathways [14, 15]. In addition, high glucose levels owing to glucose intolerance induce high levels of intracellular glucose, facilitating breast cancer and CRC cell growth [6, 8]. Thus, impaired glucose metabolism, such as IR, leading to hyperglycemia and hyperinsulinemia, contributes to overexpression of these receptors and multiple abnormal cellular signaling cascades, and therefore may be associated with carcinogenesis. Considering the relationships of these glycemic phenotypes and cancer risk, the glucose metabolism-related genetic variants that are related to impaired glucose metabolic syndromes (e.g. high glucose, insulin, and HOMA-IR levels) are plausibly associated with increased risk of breast cancer and CRC. A limited number of population-based epidemiologic studies have been performed to examine these relationships [16–22].

Breast cancer, particularly in postmenopausal women, and CRC risk are elevated among those who are obese [4, 23–26]. Obesity status and obesity-related lifestyle factors are accompanied by elevated glucose metabolism traits (e.g., insulin, glucose, and HOMA-IR levels) [4, 23, 24]. Specifically, physical inactivity and high-fat diet, as modifiable factors for obesity, [3] increase insulin levels and IR, and are associated with increased risk of breast cancer [8, 27, 28] and CRC [29–32]. Further, previous in vitro studies have revealed obesity– glucose metabolism-related gene signature–breast cancer or CRC risk pathways, suggesting that glucose metabolism-related genetic variants interact with obesity and jointly influence cancer susceptibility [15, 27, 33–36].

**Fig. 1:**
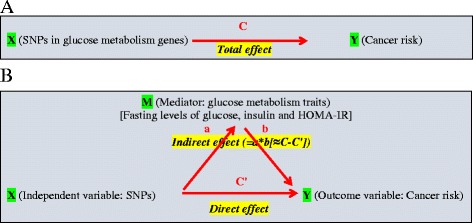
Diagrams of total, direct, and indirect pathways of SNPs in glucose metabolism genes, glucose metabolism traits, and cancer risk. (HOMA-IR, homeostatic model assessment–insulin resistance; HR, hazard ratio; SNP, single-nucleotide polymorphism.). **a** *C* is a total effect (overall genetic effect, without considering the effect of glucose metabolism traits), expressed via HR. **b** *C*′ is a direct effect (cancer risk associated with glucose metabolism-relevant genetic variants through pathways other than glucose metabolism traits), expressed via HR after accounting for mediator; a*b (*≈C-C′*) is an indirect effect (cancer risk associated with glucose metabolism-relevant genetic variants through pathways mediated by glucose metabolism traits)

In this study among postmenopausal women, we examined the pathway of glucose metabolism genetic variants, glucose metabolism traits (fasting insulin, glucose, and HOMA-IR levels), and cancer risk. We focused on the mediation effects relating glucose metabolism genetic variants (exposure) and breast cancer and CRC risk (outcome), and on the role of glucose metabolism traits (mediator) that play in this association (Fig. 1). We first evaluated the magnitude of the total effect of glucose metabolism genetic variants on breast cancer and CRC (i.e. the overall genetic effect, without considering the effect of glucose metabolism traits). We then evaluated how this total effect is partitioned into direct (cancer risk associated with glucose metabolism genetic variants through pathways other than glucose metabolism traits) and indirect effects (cancer risk associated with glucose metabolism genetic variants through pathways mediated by glucose metabolism traits). This approach allowed us to test the hypothesis that glucose metabolism-related genetic variants are associated with increased risk of cancers and that the relationships depend on impaired glucose metabolism symptoms (high insulin, glucose, and HOMA-IR levels).

Given that the association between glucose-metabolism genetic factors and glucose-metabolism traits could be influenced by obesity [4, 8, 23, 24, 27–32], and through this glycemic mechanism, obesity status and related factors are associated with breast cancer and CRC [15, 27, 33–36], we evaluated how the pathway of glucose metabolism genetic factors, glucose metabolism traits, and cancer is influenced by obesity and obesity-related factors. We examined whether glucose metabolism genetic variants’ interactions with obesity and relevant lifestyle factors influence glucose metabolism traits and whether these changes in traits alter the association between glucose metabolism traits and cancer risk. Further, we assessed whether these altered relationships (glucose metabolism gene–glucose metabolism traits relationship and glucose metabolism traits–cancer risk relationship) influence the association between glucose metabolism genetic variants and cancer risk.

Disentangling these complicated gene–phenotype–lifestyle interactions will provide insights into the role of glucose intolerance in the development of obesity-related breast cancer and CRC and suggest strategies to reduce cancer risk in postmenopausal women.

## Methods

### Study population

This study included data from 5379 participants enrolled in the Women’s Health Initiative (WHI) Harmonized and Imputed Genome-Wide Association Studies (GWAS), which is the effort of a joint imputation and harmonization effort for GWAS within the WHI Clinical Trials and Observational Studies. Details of this study’s rationale and design have been described elsewhere [37, 38]. Briefly, WHI study participants were recruited from 40 clinical centers nationwide between October 1, 1993, and December 31, 1998. Eligible women were 50–79 years old, postmenopausal, expected to live near the clinical centers for at least 3 years after enrollment, and able to provide written consent. For our study, we included only European-American women. From among the 7835 women who did not have diabetes mellitus (DM) at enrollment or later, and had at least 8 hours’ fasting glucose and/or insulin concentrations available at baseline, we excluded women who had been followed up for less than 1 year or those diagnosed with any cancer at enrollment, resulting in 6748 participants. We excluded another 1369 women whose information on covariates was unavailable, leaving a final total of 5379 women (80% of the eligible 6748). This study was approved by the institutional review boards at the University of California, Los Angeles.

### Data collection and outcome variables

Standardized written protocols had been used and periodic quality assurance performed by the WHI coordinating center to ensure uniform data collection. At baseline, participants had completed self-questionnaires on demographic and lifestyle factors and their medical and reproductive histories. Anthropometric measurements, including height, weight, and waist and hip circumferences had been obtained at baseline by trained staff. Of 33 variables initially chosen from a literature review for their associations with glucose metabolism and breast cancer and CRC, we selected 29 final variables (Table 1) for this study after performing univariate and stepwise regression analyses and multicollinearity testing.

**Table 1:** Characteristics of participants, stratified by obesity (measured via BMI)

Characteristic	Non-obese group (BMI < 30.0)		Obese group (BMI ≥ 30.0)	
	(*n* = 3675)		(*n* = 1704)	
	n	(%)	n	(%)
Age in years, median (range)	68	(50–79)	67	(50–71)*
Education				
≤ High school	1272	(34.6)	701	(41.1)*
> High school	2403	(65.4)	1003	(58.9)
Family history of diabetes mellitus				
No	2714	(73.9)	1122	(65.8)*
Yes	961	(26.1)	582	(34.2)
Family history of cancer				
No	1327	(36.1)	567	(33.3)
Yes	2348	(63.9)	1137	(66.7)
Family history of breast cancer				
No	3070	(83.5)	1428	(83.8)
Yes	605	(16.5)	276	(16.2)
Family history of colorectal cancer				
No	3106	(84.5)	1430	(83.9)
Yes	569	(15.5)	274	(16.1)
Cardiovascular disease ever				
No	3161	(86.0)	1430	(83.9)
Yes	514	(14.0)	274	(16.1)
Hypertension ever				
No	2702	(73.5)	1026	(60.2)*
Yes	973	(26.5)	678	(39.8)
High cholesterol requiring pills ever				
No	3178	(86.5)	1460	(85.7)
Yes	497	(13.5)	244	(14.3)
Smoking status				
Never	1882	(51.2)	897	(52.6)*
Past	1498	(40.8)	705	(41.4)
Current	295	(8.0)	102	(6.0)
Lifetime partner				
Have never had sex	56	(1.5)	38	(2.2)
Have had sex	3619	(98.5)	1666	(97.8)
Depressive symptom^a^				
< 0.06	3392	(92.3)	1556	(91.3)
≥ 0.06	283	(7.7)	148	(8.7)
METs·hour·week^-1 b^				
< 10	1875	(51.0)	1160	(68.1)*
≥ 10	1800	(49.0)	544	(31.9)
Total HEI-2005 score, median (range)^c^	68.7	(25.8–90.8)	65.7	(27.9–91.2)*
Dietary total sugars in g, median (range)	91.6	(4.6–350.2)	94.9	(10.8–474.5)*
Dietary alcohol per day in g, median (range)	1.506	(0.0–106.7)	0.561	(0.0–148.6)*
% calories from fat^d^				
< 40%	3057	(83.2)	1268	(74.4)*
≥ 40%	618	(16.8)	436	(25.6)
Waist circumference in cm, median (range)	81.0	(37.5–177.0)	100.0	(69.0–191.8)*
Waist-to-hip ratio, median (range)	0.798	(0.341–1.893)	0.849	(0.633–1.696)*
Oral contraceptive use				
Never	2448	(66.6)	1123	(65.9)
Ever	1227	(33.4)	581	(34.1)
History of hysterectomy or oophorectomy				
No	2457	(66.9)	1006	(59.0)*
Yes	1218	(33.1)	698	(41.0)
Age at menarche in years, median (range)	13	(≤ 9–≥ 17)	12	(≤ 9–≥ 17)*
Age at menopause in years, median (range)	50	(20–60)	50	(21–60)
Pregnancy history				
No	276	(7.5)	134	(7.9)
Yes	3399	(92.5)	1570	(92.1)
Breastfeeding at least one month				
No	1649	(44.9)	812	(47.7)
Yes	2026	(55.1)	892	(52.3)
Exogenous estrogen use				
No	2224	(60.5)	1122	(65.8)*
Yes	1451	(39.5)	582	(34.2)
Glucose in mg/dl, median (range)	92.0	(39.0–369.0)	97.0	(62.0–347.0)*
Insulin in μIU/ml, median (range)	5.5	(0.5–119.4)	9.8	(0.3–57.0)*
HOMA-IR, median (range)	1.3	(0.1–25.1)	2.4	(0.1–42.3)*

*BMI* body mass index, *HEI-2005* Healthy Eating Index-2005, *HOMA-IR* homeostatic model assessment–insulin resistance, *MET* metabolic equivalent

*p < 0.05, chi-squared or Wilcoxon’s rank-sum test

^a^Depression scales were estimated by using a short form of the Center for Epidemiologic Studies Depression Scale and categorized with 0.06 as the cutoff to detect depressive disorders

^b^Physical activity was estimated from recreational physical activity combining walking and mild, moderate, and strenuous physical activity

^c^HEI-2005 is a measure of diet quality that assesses adherence to the U.S. Department of Agriculture’s Dietary Guidelines for Americans. The total HEI score ranges from 0 to 100, with higher scores indicating higher diet quality

^d^Participants were stratified by high-fat diet using 40% as a cutoff value relevant to glucose intolerance [47]

Cancer outcomes were determined via a centralized review of medical charts, and cancer cases were coded according to the National Cancer Institute’s Surveillance, Epidemiology, and End-Results guidelines [39]. The outcome variables were the specific cancer type (breast cancer and CRC) and the time to develop such cancer. The time from enrollment to cancer development, censoring, or study end-point was recorded as the number of days and then converted into years.

### Genotyping and laboratory methods

The WHI imputed GWAS comprises six substudies (Hip Fracture GWAS, SHARe, GARNET, WHIMS, GECCO, and MOPMAP) within the WHI study. Participants provided DNA samples at baseline and genotyping included alignment (“flipping”) to the same reference panel and imputation via the 1000 Genomes reference panels. Single-nucleotide polymorphisms (SNPs) for harmonization were checked for pairwise concordance among all samples in the substudies. Initial quality assurance was conducted according to a standardized protocol, with a missing call rate of <2% and Hardy-Weinberg Equilibrium of *p* ≥ 10^−4^. Sixteen SNP candidates, available for this study with 97% R-squared imputation quality scores, were selected on the basis of their association (*p* < 5 × 10^−8^) with fasting glucose and/or insulin concentrations in a previous meta-analysis with independent replication [40–42].

Fasting blood samples had been collected from each participant at baseline by trained phlebotomists and immediately centrifuged and stored at −70 °C. Serum glucose was measured using the hexokinase method on a Hitachi 747 analyzer (Boehringer Mannheim Diagnostics), with coefficient of variation of 1.6% and correlation coefficient of values of 0.99. Serum insulin testing had been conducted by Sandwich Immunoassay on a Roche Elecsys 2010 analyzer (Roche Diagnostics). The coefficient of variation and correlation coefficient of values for insulin were 4.9% and 0.99, respectively. HOMA-IR was estimated as glucose (unit: mg/dl) × insulin (unit: μIU/ml) / 405 [43].

### Statistical analysis

Participants’ differences in baseline characteristics, stratified by obesity status (body mass index [BMI], waist circumference, and waist-to-hip ratio [w/h]), level of physical activity, and dietary fat intake, were assessed by using unpaired two-sample *t* tests for continuous variables, and chi-squared tests for categorical variables. If continuous variables were skewed or had outliers, Wilcoxon’s rank-sum test was implemented. With the regression assumptions met, multiple linear regression was performed to produce effect sizes and 95% confidence intervals (CIs) of the exposure (glucose metabolism-related SNPs with an additive and dominant model) to predict the outcomes (fasting glucose, insulin, and HOMA-IR levels) (Additional file 1: Tables S1.1–6).

The Cox proportional hazards regression model was used to obtain hazard ratios (HRs) and 95% CIs for glucose, insulin, and HOMA-IR levels and glucose metabolism-related SNPs in predicting breast cancer and CRC. The proportional hazards assumption was tested via a Schoenfeld residual plot and rho. The model was adjusted for covariates (e.g., age, education, family history of DM and cancer, comorbidity, lifestyle factors including smoking, physical activity, depression, lifetime partner, and diet, obesity, and reproductive history).

A direct and total effect size of glucose metabolism-related SNP (exposure) on breast cancer and CRC (outcome) was produced from the HR for glucose metabolism-related SNP on cancer in the Cox model that included all covariates, with (direct) and without (total) glucose, insulin, and HOMA-IR levels (mediator). The mediation effect size and testing for its significance (i.e. the pathway of glucose metabolism-SNPs and cancer risk through insulin, glucose, and HOMA-IR levels) were produced via the use of two complementary statistical methods [44–46]: 1) bootstrapping the sampling distribution for standard errors using Mplus software and 2) the percentage change in the HRs by comparing a model that includes all covariates with a model that includes all covariates and the mediator [44, 45]. These two approaches, differently from traditional Baron-Kenny steps, enabled us not only to prevent results from being affected by Type II errors but also to estimate the amount and test the significance of the mediation effect [44]. To evaluate the role of obesity and correlated lifestyle factors as an effect modifier on the pathway of glucose metabolism genetic factors, glucose metabolism traits, and cancer, we stratified participants by those potential effect modifiers, and within the strata, compared the proportions of the cancer risk contributed by glucose metabolism genetic variants through the glucose metabolism traits (indirect effect) and non-glucose metabolism pathways (direct effect). A two-tailed *p*-value <0.05 was considered statistically significant. The R statistical package (v 2.15.1) was used.

## Results

Participants’ baseline characteristics between non-obese (BMI <30.0) and obese (BMI ≥30.0) women are presented in Table 1. Obese women were younger, less educated, and more likely to have a history of hypertension and a family history of DM than non-obese women. Also obese women were less likely to be current smokers, and to meet the physical activity and dietary guidelines, and they were more likely to have higher percentages of calories from dietary fat intake. Further, more obese women tended to have a history of hysterectomy or oophorectomy and earlier menarche, and they were less likely to use exogenous estrogen. They also had higher serum levels of fasting glucose, insulin, and HOMA-IR. We stratified participants by waist circumference, w/h, level of physical activity, and dietary fat intake, using a cutoff value relevant to glucose intolerance, [47] and compared their characteristics (Additional file 2: Tables S2.1–4). The participants had been followed up through August 29, 2014 (a median follow-up period of 16 years), resulting in 326 participants (5% of non-obese and 8% of obese women) diagnosed with breast cancer, and 364 participants (6% of non-obese and 8% of obese women) diagnosed with CRC.

Sixteen SNPs were selected from previous GWAS as being associated with glucose metabolism traits. The allele frequencies of these SNPs in our population were consistent with frequencies of those in a European population [48]. No significant differences in allele frequency between strata (obesity, physical activity, and high-fat diet) were observed (Additional file 3: Tables S3.1–5).

### Breast cancer risk associated with glucose metabolism-related SNPs mediated through glucose metabolism traits, stratified by obesity status (BMI, waist, and w/h), level of physical activity, and dietary fat intake

We partitioned the total effect of glucose metabolism-related SNPs on breast cancer risk into indirect (via glucose metabolism traits) and direct (not via glucose metabolism traits) effects. Each of these analyses was mediated by fasting glucose (Table 2), HOMA-IR (Table 3), and insulin levels (Additional file 4: Table S4.1). For each mediator, the glucose metabolism-SNP–cancer association was evaluated, stratified by obesity status (BMI < 30 vs. ≥ 30; waist ≤88 cm vs. > 88 cm; and w/h ≤ 0.85 vs. > 0.85), level of physical activity (metabolic equivalent [MET] ≥ 10 vs. < 10), and dietary fat intake (< 40% vs. ≥ 40% calories from fat).

**Table 2:** Mediation effect of glucose on the relationship between glucose metabolism–relevant SNPs and breast cancer risk, stratified by obesity status and obesity-related factors

SNP	Nearest gene	Effect allele/Other allele	Favorable Energy Balance Group						Unfavorable Energy Balance Group					
			Direct effect		Indirect effect		Total effect		Direct effect		Indirect effect		Total effect	
			Breast cancer risk in relation to SNP through pathways other than *glucose*		Breast cancer risk in relation to SNP through *glucose*		Breast cancer risk in relation to SNP		Breast cancer risk in relation to SNP through pathways other than *glucose*		Breast cancer risk in relation to SNP through *glucose*		Breast cancer risk in relation to SNP	
			HR^a^	95% CI	Effect size^a^	95% CI	HR^a^	95% CI	HR^a^	95% CI	Effect size^a^	95% CI	HR^a^	95% CI
BMI^b^														
rs560887	*G6PC2*	T/C	1.11	(0.89–1.39)	0.01	(−0.04**–**0.02)	1.12	(0.90–1.39)	**1.34**	**(1.04–1.73)**	−0.004	(−0.01**–**0.02)	**1.35**	**(1.05–1.74)**
Waist^c^														
rs560887	*G6PC2*	T/C	1.09	(0.85–1.39)	0.004	(−0.02–0.01)	1.10	(0.86–1.40)	**1.35**	**(1.07–1.70)**	−0.01	(−0.01–0.02)	**1.33**	**(1.06–1.66)**
w/h Ratio^d^														
rs560887	*G6PC2*	T/C	1.07	(0.89–1.39)	0.01	(−0.03–0.01)	1.10	(0.89–1.36)	**1.47**	**(1.13–1.92)**	−0.003	(−0.003–0.01)	**1.42**	**(1.10–1.85)**
rs780094	*GCKR*	C/T	1.01	(0.82–1.23)	−0.01	(−0.03–0.01)	1.02	(0.84–1.24)	**1.33**	**(1.00–1.76)**	0.002	(−0.003–0.01)	1.22	(0.93–1.60)
rs35767	*IGF1*	A/G	0.91	(0.68–1.21)	0.002	(−0.01–0.004)	0.93	(0.70–1.23)	**1.43**	**(1.03–1.98)**	<0.001	(−0.004–0.004)	**1.48**	**(1.08–2.03)**
Dietary fat intake^e^														
rs560887	*G6PC2*	T/C	1.14	(0.95–1.37)	−0.01	(−0.003–0.02)	1.14	(0.95–1.37)	**1.56**	**(1.07–2.28)**	0.02	(−0.07–0.03)	**1.59**	**(1.10–2.31)**

*BMI* body mass index, *CI* confidence interval, *HR* hazard ratio, *SNP* single–nucleotide polymorphism, *w/h ratio* waist-to-hip ratio

*Note*: Proportions explained by glucose for SNP–breast cancer risk association for **rs560887** (8.3%,10.0%, 30.0%, and 0% among non-obese group [BMI < 30, waist ≤88 cm, w/h ≤ 0.85, and <40% calories from fat, respectively]; 2.9%, 6.1%, 11.9%, and 5.1% among obese-group [BMI ≥ 30, waist >88 cm, w/h > 0.85, and ≥40% calories from fat, respectively]), for **rs780094** (61.7% in w/h ≤ 0.85; 48.9% in w/h > 0.85), and for **rs35767** (1.9% in w/h ≤ 0.85; 9.7% in w/h > 0.85). Only SNPs having statistically significant results are included. Numbers in bold face are statistically significant

^a^Multivariate regression was adjusted by covariates (age, education, family history of diabetes mellitus, family history of breast cancer, cardiovascular disease ever, hypertension ever, high cholesterol requiring pills ever, total Healthy Eating Index-2005 score, dietary alcohol and total sugars per day, smoking status, lifetime partner, depressive symptom, oral contraceptive use, history of hysterectomy or oophorectomy, age at menarche, age at menopause, pregnancy history, breastfeeding at least 1 month, and hormone therapy); effect-modifier variables (physical activity, BMI, and w/h ratio), when not evaluated as effect modifier variables, were adjusted as a covariate; when stratified via waist circumference, w/h ratio was not adjusted

^b^Participants stratified by BMI as non-obese (BMI < 30, *n* = 3675) or obese (BMI ≥ 30, *n* = 1704); interaction test presented for the effect of BMI on the association between breast cancer and rs560887 (effect size −0.38, *p*-value 0.28)

^c^Participants stratified by waist circumference as non-obese (waist ≤88 cm; *n* = 3042) or obese (waist >88 cm; *n* = 2337); interaction test presented for the effect of waist circumference on the association between breast cancer and rs560887 (effect size −0.55, *p*-value 0.13)

^d^Participants stratified by w/h as non-obese (w/h ≤ 0.85; *n* = 3712) or obese (w/h > 0.85; *n* = 1667); interaction tests presented for the effect of w/h on the association between breast cancer and rs560887 (effect size −0.57, *p*-value 0.11), rs780094 (effect size 0.58, *p*-value 0.08), and rs35767 (effect size 0.54, *p*-value 0.01)

^e^Participants stratified by dietary fat intake as non-obese (< 40% calories from fat; *n* = 4325) or obese (≥ 40% calories from fat; *n* = 1054); interaction test presented for the effect of dietary fat intake on the association between breast cancer and rs560887 (effect size −0.45, *p*-value 0.26)

**Table 3:** Mediation effect of HOMA-IR on the relationship between glucose metabolism–relevant SNPs and breast cancer risk, stratified by obesity status and obesity-related factors

SNP	Nearest gene	Effect allele/Other allele	Favorable Energy Balance Group						Unfavorable Energy Balance Group					
			Direct effect		Indirect effect		Total effect		Direct effect		Indirect effect		Total effect	
			Breast cancer risk in relation to SNP through pathways other than *HOMA-IR*		Breast cancer risk in relation to SNP through *HOMA-IR*		Breast cancer risk in relation to SNP		Breast cancer risk in relation to SNP through pathways other than *HOMA-IR*		Breast cancer risk in relation to SNP through *HOMA-IR*		Breast cancer risk in relation to SNP	
			HR^a^	95% CI	Effect size^a^	95% CI	HR^a^	95% CI	HR^a^	95% CI	Effect size^a^	95% CI	HR^a^	95% CI
BMI^b^														
rs560887	*G6PC2*	T/C	1.17	(0.93–1.46)	<0.001	(−0.003**–**0.003)	1.12	(0.90–1.39)	**1.35**	**(1.04–1.76)**	<0.001	(−0.02**–**0.02)	**1.35**	**(1.05–1.74)**
Waist^c^														
rs560887	*G6PC2*	T/C	1.11	(0.86–1.42)	−0.003	(−0.01–0.01)	1.10	(0.86–1.40)	**1.39**	**(1.10–1.77)**	−0.002	(−0.01–0.01)	**1.33**	**(1.06–1.66)**
Waist/hip Ratio^d^														
rs560887	*G6PC2*	T/C	1.11	(0.72–1.12)	0.001	(−0.01–0.004)	1.10	(0.89–1.36)	**1.50**	**(1.14–1.97)**	−0.001	(−0.01–0.01)	**1.42**	**(1.10–1.85)**
rs35767	*IGF1*	A/G	0.92	(0.69–1.24)	0.001	(−0.01–0.003)	0.93	(0.70–1.23)	**1.42**	**(1.02–1.98)**	−0.002	(−0.01–0.01)	**1.48**	**(1.08–2.03)**
Dietary fat intake^e^														
rs560887	*G6PC2*	T/C	1.16	(0.96–1.41)	0.01	(−0.01–0.003)	1.14	(0.95–1.37)	**1.59**	**(1.08–2.34)**	0.003	(−0.02–0.02)	**1.59**	**(1.10–2.31)**

*BMI* body mass index, *CI* confidence interval, *HOMA-IR* homeostatic model assessment–insulin resistance, *HR* hazard ratio, *SNP* single–nucleotide polymorphism, *w/h ratio* waist-to-hip ratio

*Note*: Proportions explained by HOMA-IR for SNP–breast cancer risk association for **rs560887** (41.7%,10%, 10%, and 14.3% among non-obese group [BMI < 30, waist ≤88 cm, w/h ≤ 0.85, and <40% calories from fat, respectively]; 0%, 18.1%, 19.1%, and 0% among obese-group [BMI ≥ 30, waist >88 cm, w/h > 0.85, and ≥40% calories from fat, respectively]), and for **rs35767** (0.4% in w/h ≤ 0.85; 11.9% in w/h > 0.85). Only SNPs having statistically significant results are included. Numbers in bold face are statistically significant

^a^Multivariate regression was adjusted by covariates (age, education, family history of diabetes mellitus, family history of breast cancer, cardiovascular disease ever, hypertension ever, high cholesterol requiring pills ever, total Healthy Eating Index-2005 score, dietary alcohol and total sugars per day, smoking status, lifetime partner, depressive symptom, oral contraceptive use, history of hysterectomy or oophorectomy, age at menarche, age at menopause, pregnancy history, breastfeeding at least 1 month, and hormone therapy); effect-modifier variables (physical activity, BMI, and w/h ratio), when not evaluated as effect modifier variables, were adjusted as a covariate; when stratified via waist circumference, w/h ratio was not adjusted

^b^Participants stratified by BMI as non-obese (BMI < 30, *n* = 3675) or obese (BMI ≥ 30, *n* = 1704); interaction test presented for the effect of BMI on the association between breast cancer and rs560887 (effect size −0.38, *p*-value 0.28)

^c^Participants stratified by waist circumference as non-obese (waist ≤88 cm; *n* = 3042) or obese (waist >88 cm; *n* = 2337); interaction test presented for the effect of waist circumference on the association between breast cancer and rs560887 (effect size −0.55, *p*-value 0.13)

^d^Participants stratified by w/h as non-obese (w/h ≤ 0.85; *n* = 3712) or obese (w/h > 0.85; *n* = 1667); interaction tests presented for the effect of w/h on the association between breast cancer and rs560887 (effect size −0.57, *p*-value 0.11) and rs35767 (effect size 0.54, *p*-value 0.01)

^e^Participants stratified by dietary fat intake as non-obese (< 40% calories from fat; *n* = 4325) or obese (≥ 40% calories from fat; *n* = 1054); interaction test presented for the effect of dietary fat intake on the association between breast cancer and rs560887 (effect size −0.45, *p*-value 0.26)

Of the 16 candidate SNPs, three had significant associations with breast cancer risk. The SNP–cancer risk effect was stronger in each SNP for a direct effect than an indirect effect regardless of the mediator. Carriers of the *G6PC2* rs560887 T minor-allele were associated with increased breast cancer risk in obese women, stratified by BMI, waist, w/h, and dietary fat intake (Tables 2 and 3, and Additional file 4: Table S4.1). Roughly 15% of the breast cancer risk owing to this genetic variant was mediated via glucose metabolism traits in the obese group; no significant differences in mediation effect were found between the obese and non-obese women.

Carriers of the *IGF1* rs35767 A minor-allele had associations similar to those found in the carriers of *G6PC2* (Tables 2 and 3, and Additional file 4: Table S4.1). Compared with the carriers in the non-obese group (w/h ≤ 0.85), in whom no significant association with cancer was found, the carriers in the obese group (w/h > 0.85) had an association with increased breast cancer risk; further, in this obese group, about 10% of the breast cancer risk associated with this genetic variant was dependent on glucose metabolism traits. In addition, no differences were apparent in mediation effect between women with w/h ≤ 0.85 and those with w/h > 0.85. Carriers of the *GCKR* rs780094 C major-allele had an association with increased risk of breast cancer in women with w/h > 0.85 (Table 2); approximately 50% of cancer risk attributable to this variant was mediated via glucose levels in this obese group.

### CRC risk associated with glucose metabolism-related SNPs mediated through glucose metabolism traits, stratified by obesity status (BMI, waist, and w/h), level of physical activity, and dietary fat intake

We also split the total effect of the CRC risk–glucose metabolism SNP relationship into direct and indirect effects through fasting glucose (Table 4), HOMA-IR (Table 5), and insulin levels (Additional file 4: Table S4.2). For each mediator, those effects were stratified by obesity status (BMI, waist, and w/h), level of physical activity, and dietary fat intake. Overall, the direct effect of glucose metabolism SNPs on increased CRC risk accounted for a majority of the total effect, suggesting a minimal influence of indirect effect on the total effect. In addition, the indirect effects mediated via glucose metabolism traits were not apparently different between obesity strata.

**Table 4:** Mediation effect of glucose on the relationship between glucose metabolism–relevant SNPs and CRC risk, stratified by obesity status and obesity-related factors

SNP	Nearest gene		Favorable Energy Balance Group						Unfavorable Energy Balance Group					
			Direct effect		Indirect effect		Total effect		Direct effect		Indirect effect		Total effect	
		Effect allele/Other allele	CRC risk in relation to SNP through pathways other than *glucose*		CRC risk in relation to SNP through *glucose*		CRC risk in relation to SNP		CRC risk in relation to SNP through pathways other than *glucose*		CRC risk in relation to SNP through *glucose*		CRC risk in relation to SNP	
			HR^a^	95% CI	Effect size^a^	95% CI	HR^a^	95% CI	HR^a^	95% CI	Effect size^a^	95% CI	HR^a^	95% CI
BMI^b^														
rs4607517	*GCK*	G/A	0.79	(0.60–1.02)	**0.02**	**(0.002–0.04)**	**0.80**	**(0.64–1.00)**	1.00	(0.65–1.55)	0.001	(−0.01**–**0.01)	1.06	(0.75–1.51)
rs174550	*FADS1*	T/C	1.15	(0.92–1.43)	−0.002	(−0.01**–**0.01)	1.05	(0.87–1.27)	1.40	(0.97–2.03)	<0.001	(−0.01**–**0.01)	**1.37**	**(1.02–1.83)**
rs11605924	*CRY2*	C/A	0.89	(0.73–1.09)	−0.004	(−0.01**–**0.02)	**0.82**	**(0.69–0.98)**	1.32	(0.94–1.85)	<0.001	(−0.004**–**0.004)	1.07	(0.82–1.39)
Waist^c^														
rs560887	*G6PC2*	T/C	**0.75**	**(0.58**–**0.99)**	0.01	(−0.03**–**0.004)	0.88	(0.70**–**1.11)	1.00	(0.75**–**1.34)	0.01	(−0.04**–**0.02)	1.01	(0.80**–**1.27)
rs174550	*FADS1*	T/C	1.17	(0.91–1.51)	−0.01	(−0.02–0.01)	1.03	(0.83–1.28)	1.23	(0.93–1.63)	0.01	(−0.01–0.02)	**1.25**	**(1.00–1.57)**
rs11605924	*CRY2*	C/A	0.84	(0.67–1.06)	−0.01	(−0.01–0.02)	**0.81**	**(0.66–0.99)**	1.20	(0.92–1.56)	0.004	(−0.02–0.01)	0.97	(0.79–1.20)
w/h Ratio^d^														
rs10885122	*ADRA2A*	G/T	0.80	(0.59–1.07)	−0.002	(−0.01–0.01)	0.84	(0.64–1.08)	1.36	(0.81–2.30)	<0.001	(−0.003–0.003)	**1.58**	**(1.01–2.48)**
Physical activity level^e^														
rs4607517	*GCK*	G/A	0.73	(0.52–1.00)	0.01	(−0.01–0.04)	**0.72**	**(0.55–0.95)**	0.95	(0.70–1.31)	0.01	(−0.01–0.02)	0.99	(0.76–1.28)
Dietary fat intake^f^														
rs4607517	*GCK*	G/A	0.91	(0.71–1.17)	0.01	(−0.004–0.03)	0.94	(0.76–1.17)	0.57	(0.33–1.01)	−0.003	(−0.01–0.01)	**0.66**	**(0.43–1.00)**
rs11558471	*SLC30A8*	A/G	1.07	(0.87–1.31)	−0.02	(−0.04–0.004)	0.95	(0.80–1.12)	1.60	(0.93–2.75)	−0.002	(−0.01–0.01)	**1.60**	**(1.07–2.40)**

*BMI* body mass index, *CI* confidence interval, *CRC* colorectal cancer, *HR* hazard ratio, *SNP* single–nucleotide polymorphism, *w/h ratio* waist-to-hip ratio

*Note*: Proportions explained by glucose for SNP–CRC risk association for **rs4607517** (1.5%, 0.5%, and 3.3% among non-obese group [BMI < 30, MET ≥10, and <40% calories from fat, respectively]; N/A [> 100%], 3.3%, and 12.5% among obese-group [BMI ≥ 30, MET <10, and ≥40% calories from fat, respectively]), for **rs174550** (N/A [> 100%] and N/A [> 100%] among non-obese group [BMI < 30 and waist ≤88 cm, respectively]; 9.9% and 10.3% among obese-group [BMI ≥ 30 and waist >88 cm, respectively]), for **rs11605924** (8.5% and 3.7% among non-obese group [BMI < 30 and waist ≤88 cm, respectively]; N/A [> 100%] and N/A [> 100%] among obese-group [BMI ≥ 30 and waist >88 cm, respectively]), for **rs560887** (14.7% in waist ≤88 cm; N/A [>100%] in waist >88 cm), for **rs10885122** (4.7% in w/h ≤ 0.85; 37.7% in w/h > 0.85), and for **rs11558471** (12.5% in <40% calories from fat; 0.9% in ≥40% calories from fat). Only SNPs having statistically significant results are included. Numbers in bold face are statistically significant

^a^Multivariate regression was adjusted by covariates (age, education, family history of diabetes mellitus, family history of colorectal cancer, cardiovascular disease ever, hypertension ever, high cholesterol requiring pills ever, total Healthy Eating Index-2005 score, dietary alcohol and total sugars per day, smoking status, lifetime partner, depressive symptom, oral contraceptive use, history of hysterectomy or oophorectomy, age at menarche, age at menopause, pregnancy history, breastfeeding at least 1 month, and hormone therapy); effect-modifier variables (physical activity, BMI, and w/h ratio), when not evaluated as effect modifier variables, were adjusted as a covariate; when stratified via waist circumference, w/h ratio was not adjusted

^b^Participants stratified by BMI as non-obese (BMI < 30, *n* = 3675) or obese (BMI ≥ 30, *n* = 1704); interaction tests presented for the effect of BMI on the association between CRC and rs4607517 (effect size −0.30, *p*-value 0.65), rs174550 (effect size −0.70, *p*-value 0.09), and rs11605924 (effect size −0.38, *p*-value 0.15)

^c^Participants stratified by waist circumference as non-obese (waist ≤88 cm; *n* = 3042) or obese (waist >88 cm; *n* = 2337); interaction tests presented for the effect of waist circumference on the association between CRC and rs560887 (effect size −0.40, *p*-value 0.27), rs174550 (effect size −0.89, *p*-value 0.01), and rs11605924 (effect size −0.42, *p*-value 0.09)

^d^Participants stratified by w/h as non-obese (w/h ≤ 0.85; *n* = 3712) or obese (w/h > 0.85; *n* = 1667); interaction test presented for the effect of w/h on the association between CRC and rs10885122 (effect size 0.59, *p*-value 0.02)

^e^Participants stratified by physical activity level as non-obese (MET ≥10; *n* = 2344) or obese (MET <10; *n* = 3035); interaction test presented for the effect of physical activity on the association between CRC and rs4607517 (effect size 0.94, *p*-value 0.14)

^f^Participants stratified by dietary fat intake as non-obese (< 40% calories from fat; *n* = 4325) or obese (≥ 40% calories from fat; *n* = 1054); interaction tests presented for the effect of dietary fat intake on the association between CRC and rs4607517 (effect size 1.45, *p*-value 0.01) and rs11558471 (effect size −1.53, *p*-value 0.04)

**Table 5:** Mediation effect of HOMA-IR on the relationship between glucose metabolism–relevant SNPs and CRC risk, stratified by obesity status and obesity-related factors

SNP	Nearest gene	Effect allele/ Other allele	Favorable Energy Balance Group						Unfavorable Energy Balance Group					
			Direct effect		Indirect effect		Total effect		Direct effect		Indirect effect		Total effect	
			CRC risk in relation to SNP through pathways other than *HOMA-IR*		CRC risk in relation to SNP through *HOMA-IR*		CRC risk in relation to SNP		CRC risk in relation to SNP through pathways other than *HOMA-IR*		CRC risk in relation to SNP through *HOMA-IR*		CRC risk in relation to SNP	
			HR^a^	95% CI	Effect size^a^	95% CI	HR^a^	95% CI	HR^a^	95% CI	Effect size^a^	95% CI	HR^a^	95% CI
BMI^b^														
rs2191349	DGKB/TMEM195	G/T	1.04	(0.84–1.28)	<0.001	(−0.004**–**0.004)	1.07	(0.90–1.28)	**1.45**	**(1.03–2.03)**	<0.001	(−0.003**–**0.003)	1.17	(0.90–1.52)
rs4607517	*GCK*	G/A	0.79	(0.61–1.03)	<0.001	(−0.01–0.01)	**0.80**	**(0.64–1.00)**	1.04	(0.66–1.62)	<0.001	(−0.004**–**0.003)	1.06	(0.75–1.51)
rs174550	*FADS1*	T/C	1.16	(0.93–1.45)	<0.001	(−0.001**–**0.001)	1.05	(0.87–1.27)	1.40	(0.96–2.04)	−0.01	(−0.02**–**0.01)	**1.37**	**(1.02–1.83)**
rs11605924	*CRY2*	C/A	0.87	(0.71–1.07)	<0.001	(−0.01–0.01)	**0.82**	**(0.69–0.98)**	1.32	(0.93–1.87)	<0.001	(−0.003**–**0.003)	1.07	(0.82–1.39)
Waist^c^														
rs2191349	DGKB/TMEM195	G/T	0.97	(0.76–1.23)	0.002	(−0.01–0.01)	1.00	(0.81–1.23)	**1.37**	**(1.05–1.79)**	−0.002	(−0.004–0.01)	1.20	(0.97–1.48)
rs174550	*FADS1*	T/C	1.22	(0.94–1.58)	−0.002	(−0.01–0.01)	1.03	(0.83–1.28)	1.24	(0.93–1.65)	−0.01	(−0.02–0.002)	**1.25**	**(1.00–1.57)**
rs11605924	*CRY2*	C/A	0.84	(0.67–1.06)	<0.001	(−0.01–0.01)	**0.81**	**(0.66–0.99)**	1.17	(0.90–1.54)	<0.001	(−0.01–0.01)	0.97	(0.79–1.20)
Waist/hip Ratio^d^														
rs2191349	DGKB/TMEM195	G/T	1.04	(0.84–1.29)	0.003	(−0.01–0.004)	1.03	(0.86–1.23)	**1.38**	**(1.00–1.89)**	<0.001	(−0.01–0.01)	1.24	(0.97–1.59)
rs10885122	*ADRA2A*	G/T	0.78	(0.58–1.05)	<0.001	(−0.01–0.01)	0.84	(0.64–1.08)	1.31	(0.77–2.20)	<0.001	(−0.01–0.01)	**1.58**	**(1.01–2.48)**
rs174550	*FADS1*	T/C	1.15	(0.92–1.45)	0.002	(−0.004–0.01)	1.08	(0.89–1.30)	**1.43**	**(1.01–2.03)**	−0.008	(−0.02–0.004)	1.27	(0.97–1.66)
Physical activity level^e^														
rs4607517	*GCK*	G/A	0.73	(0.52–1.01)	<0.001	(−0.003–0.003)	**0.72**	**(0.55–0.95)**	0.95	(0.69–1.31)	−0.001	(−0.01–0.004)	0.99	(0.76–1.28)
Dietary fat intake^f^														
rs4607517	*GCK*	G/A	0.92	(0.72–1.18)	<0.001	(−0.01–0.004)	0.94	(0.76–1.17)	0.58	(0.33–1.03)	−0.001	(−0.01–0.001)	**0.66**	**(0.43–1.00)**
rs11558471	*SLC30A8*	A/G	1.08	(0.88–1.33)	<0.001	(−0.01–0.01)	0.95	(0.80–1.12)	1.64	(0.94–2.85)	−0.003	(−0.01–0.01)	**1.60**	**(1.07–2.40)**

*BMI* body mass index, *CI* confidence interval, *CRC* colorectal cancer, *HOMA-IR* homeostatic model assessment–insulin resistance, *HR* hazard ratio, *SNP* single–nucleotide polymorphism, *w/h ratio* waist-to-hip ratio

*Note*: Proportions explained by HOMA-IR for SNP–CRC risk association for **rs2191349** (42.8%, 2.0%, and 33.3% among non-obese group [BMI < 30, waist ≤88 cm, w/h ≤ 0.85, respectively]; N/A [> 100%], 85.0%, and 58.3% among obese-group [BMI ≥ 30, waist >88 cm, w/h > 0.85, respectively]), for **rs4607517** (0.4%, 0.7%, and 2.7% among non-obese group [BMI < 30, MET ≥10, and <40% calories from fat, respectively]; 42.8%, 3.6%, and 10.9% among obese-group [BMI ≥ 30, MET <10, and ≥40% calories from fat, respectively]), for **rs174550** (N/A [> 100%], N/A [> 100%], and N/A[> 100%] among non-obese group [BMI < 30, waist ≤88 cm, and w/h ≤ 0.85, respectively]; 8.7%, 6.3%, and 63.3% among obese-group [BMI ≥ 30, waist >88 cm, and w/h > 0.85, respectively]), for **rs11605924** (6.1% and 3.7% among non-obese group [BMI < 30 and waist ≤88 cm, respectively]; N/A [> 100%] and N/A [> 100%] among obese-group [BMI ≥ 30 and waist >88 cm, respectively]), for **rs10885122** (6.7% in w/h ≤ 0.85; 47.6% in w/h > 0.85), and for **rs11558471** (13.8% in <40% calories from fat; 5.7% in ≥40% calories from fat). Only SNPs having statistically significant results are included. Numbers in bold face are statistically significant

^a^Multivariate regression was adjusted by covariates (age, education, family history of diabetes mellitus, family history of colorectal cancer, cardiovascular disease ever, hypertension ever, high cholesterol requiring pills ever, total Healthy Eating Index-2005 score, dietary alcohol and total sugars per day, smoking status, lifetime partner, depressive symptom, oral contraceptive use, history of hysterectomy or oophorectomy, age at menarche, age at menopause, pregnancy history, breastfeeding at least 1 month, and hormone therapy); effect-modifier variables (physical activity, BMI, and w/h ratio), when not evaluated as effect modifier variables, were adjusted as a covariate; when stratified via waist circumference, w/h ratio was not adjusted

^b^Participants stratified by BMI as non-obese (BMI < 30, *n* = 3675) or obese (BMI ≥ 30, *n* = 1704); interaction tests presented for the effect of BMI on the association between CRC and rs2191349 (effect size −0.10, *p*-value 0.70), rs4607517 (effect size −0.30, *p*-value 0.65), rs174550 (effect size −0.70, *p*-value 0.09), and rs11605924 (effect size −0.38, *p*-value 0.15)

^c^Participants stratified by waist circumference as non-obese (waist ≤88 cm; *n* = 3042) or obese (waist >88 cm; *n* = 2337); interaction tests presented for the effect of waist circumference on the association between CRC and rs2191349 (effect size −0.21, *p*-value 0.38), rs174550 (effect size −0.89, *p*-value 0.01), and rs11605924 (effect size −0.42, *p*-value 0.09)

^d^Participants stratified by w/h as non-obese (w/h ≤ 0.85; *n* = 3712) or obese (w/h > 0.85; *n* = 1667); interaction tests presented for the effect of w/h on the association between CRC and rs2191349 (effect size −0.21, *p*-value 0.40), rs10885122 (effect size 0.59, *p*-value 0.02), and rs174550 (effect size −0.34, *p*-value 0.34)

^e^Participants stratified by physical activity level as non-obese (MET ≥10; *n* = 2344) or obese (MET <10; *n* = 3035); interaction test presented for the effect of physical activity on the association between CRC and rs4607517 (effect size 0.94, *p*-value 0.14)

^f^Participants stratified by dietary fat intake as non-obese (< 40% calories from fat; *n* = 4325) or obese (≥ 40% calories from fat; *n* = 1054); interaction tests presented for the effect of dietary fat intake on the association between CRC and rs4607517 (effect size 1.45, *p*-value 0.01) and rs11558471 (effect size −1.53, *p*-value 0.04)

Carriers of the *GCK* rs4607517 G major-allele had an association with decreased CRC risk in non-obese women with BMI < 30 and MET ≥10, and in obese women with ≥40% calories from fat (see total effect in Tables 4 and 5). Compared with the total effects, the direct effects of glucose metabolism-related SNP on CRC risk, after accounting for glucose (Table 4) or HOMA-IR (Table 5), decreased slightly but were no longer statistically significant; it suggested existence of glucose metabolism traits’ mediation effects (roughly, 10%) on the SNP–cancer risk. Similarly, carriers of the *CRY2* rs11605924 C major-allele had an association with decreased CRC risk in women with BMI < 30 and waist ≤88 cm (Tables 4 and 5); after accounting for glucose (Table 4) or HOMA-IR (Table 5), the direct effects were no longer significant, indicating potential mediation effects (roughly 5%) on the SNP–CRC risk association. In addition, carriers of the *G6PC2* rs560887 T minor-allele had an association with decreased CRC risk in women with waist ≤88 cm, and the mediation effect of glucose on the SNP–CRC risk association in these non-obese carriers resulted in the decreased direct effect (roughly 15%) of CRC risk in relation to *G6PC2* carriers (Table 4).

In contrast, carriers of the *FADS1* rs174550 T major-allele, the *ADRA2A* rs10885122 G major-allele, and the *SLC30A8* rs11558471 A major-allele had associations with increased CRC risk in obese women (BMI ≥ 30, waist >88 cm for *FADS1* carriers; w/h > 0.85 for *ADRA2A* carriers; and ≥40% calories from fat for *SLC30A8* carriers) (Tables 4 and 5, and Additional file 4: Table S4.2). Roughly, less than 10% of the CRC risk due to each genetic variant was mediated via glucose, HOMA-IR, or insulin in the relevant obese groups. No significantly different mediation effects were found between obesity strata. Likewise, carriers of the *DGKB/TMEM195* rs2191349 G minor-allele had an association with increased risk of CRC in obese women (BMI ≥ 30, waist >88 cm, and w/h > 0.85) (Table 5 and Additional file 4: Table S4.2). The insulin effect as a mediator in these obese carriers was minimal (15%) (Additional file 4: Table S4.2). On the contrary, the HOMA-IR mediator effect in this group (Table 5) accounted for approximately 50% of the total effect. This resulted in the elevated and significant direct effect of SNP–CRC risk (i.e. from total effect after accounting for the mediators); it suggests a positive effect of HOMA-IR on the total effect of the SNP–CRC association.

## Discussion

In this retrospective study of data from a large cohort of postmenopausal women, by using 16 glucose metabolism-related SNPs previously associated with glycemic metabolic traits, [40–42] we partitioned the total effect of glucose metabolism genetic variants on breast cancer and CRC into direct (cancer risk associated with SNPs mediated through pathways other than glucose metabolism traits) and indirect (cancer risk associated with SNPs mediated by glucose metabolism traits) effects. By stratifying data via obesity status and obesity-relevant lifestyle factors, we also assessed how those effects differed between strata. There have been relatively few population-based epidemiologic studies between glucose metabolism genetic variants and breast cancer and CRC risk [16–22]. To our knowledge, this is the first study to evaluate the association between glucose metabolism genetic variants and breast cancer and CRC risk by partitioning the glucose metabolism genetic variants’ effects on the risk for those cancers into direct and indirect effects. Additionally, we assessed the role of obesity and related factors as effect modifiers.

We found that among the16 glucose metabolism-related SNPs evaluated, three were associated with breast cancer risk, and seven with CRC risk. These SNPs’ associations with cancer risk differed between non-obese and obese carriers, indicating that glucose metabolism-related SNPs’ interactions with obesity and related lifestyle factors influence cancer risk. For most of the SNPs we studied, the direct effects on cancer risk accounted for a majority of the total effect: only roughly 15% of the cancer risk associated with glucose metabolism-related SNPs was mediated via glucose metabolism traits. This suggests that glucose metabolism traits are not the main mediators through which glucose metabolism-related SNPs are associated with increased risk for breast cancer and CRC. Further, no apparent differences in the indirect effects (mediated via glucose metabolism traits) were observed between non-obese and obese strata. Our findings thus indicate that glucose metabolism-related genetic variants interact with obesity and lifestyle factors, resulting in altered cancer risk not through glucose metabolism traits pathways, but through different mechanisms.

In relation to breast cancer risk, obese carriers of *G6PC2*, *IGF1*, and *GCKR* had an association with increased risk. Expression of the *G6PC2* gene (glycolytic inhibitor) is elevated in cancer cells and related to a decreased survival rate in cancer patients, suggesting its role in glucose metabolism and cell cycle control in cancer cells [49–51]. The *IGF1* and *GCKR* variants are related to glucose metabolism; both are highly expressed in the liver, contributing to hepatic glucose metabolism [41]. IGFI encodes insulin-like growth factor I, which is well known to increase cancer risk, and elevates HOMA-IR levels [22, 40]. Additionally, *GCKR* inhibits glucokinase, a key protein in glucose metabolism, leading to increased hepatic glucose production [41, 52]. These facts support the biological plausibility of the carriers’ association with increased breast cancer risk. Further, in this study, the carriers of these variants had association with breast cancer, but only among the obese women, suggesting that adiposity plays a strong role in modulating the effect of these variants on carcinogenesis. Interestingly, the mediation effects of glucose metabolism traits accounted for only a small portion of the overall the *G6PC2*– and *IGF1*–cancer associations in both non-obese and obese women, suggesting that different pathways exist through which obesity interacts with the G6PC2 and IGF1 genetic variants and breast cancer risk. In contrast, the *GCKR* variant’s effect on cancer was mediated through glucose by 50% in obese women (but not in non-obese women), indicating that an adiposity-related carcinogenetic pathway in this variant intermingles with the glucose-intolerance system.

Of the seven SNPs related to CRC risk, three (*GCK*, *CRY2*, and *G6PC2*) had a lower association with CRC among non-obese women. *GCK* opposing *G6PC2* encodes for glucokinsase, and mutation of this gene is related to DM and glucose metabolism; further, the *GCK* variant is associated with prostatic and pancreatic cancers [53, 54]. Our study showed a reduced CRC risk in non-obese female carriers of this variant, indicating that a cancer-specific mechanism incorporating glucose metabolism traits and genes as well as obesity should be investigated. In addition, mutation of *CRY2* results in dysfunction of circadian rhythms and is associated with tumorigenesis [20, 55]. Our finding of reduced CRC risk associated with the *CRY2* variant in non-obese women warrants further study.

The other four of the seven CRC related SNPs in our study (*FADS1*, *ADRA2A*, *SLC30A8*, and *DGKB/TMEM195*) had an increased relationship with CRC among obese women. *FADS1*, which encodes fatty acid desaturase 1, produces arachidonic acid related to increased insulin. One earlier study [19] reported CRC risk associated with this genetic variant, and their results are consistent with ours. *ADRA2A* and *SLC30A8* have not been studied for an association with CRC, but the functional changes that have been reported followed by mutations (in *ADRA2A,* modified insulin release by adrenergic suppression, and in *SLC30A8*, altered storage and maturation of insulin in beta cells [40, 56]) support our findings of increased CRC risk in relation to these variants. Finally, *DGKB* regulates diacylglycerol and potentiates insulin secretion, indicating that its mutation influences glucose homeostasis [40]; our findings suggest that this genetic variant is related to carcinogenesis in obese women.

Although obesity interacts with these seven SNPs and influences CRC risk differently between non-obese and obese carriers, the indirect effects of glucose metabolism traits on the SNP–CRC risk were minimal and did not differ between obesity strata (except in the case of *DGKB/TMEM195*). Further study is needed to examine obesity–glycemic gene–CRC mechanisms mediated through different pathways. In contrast, among obese women, roughly 50% of CRC risk associated with *DGKB/TMEM195* variant was mediated via HOMA-IR. This supports the role of adiposity in carcinogenesis through deregulated glycemic metabolism.

We did not conduct any subtype analyses of breast cancer cases due to insufficient statistical power (cases represented less than 3% of each subset). Since we were using this analysis to generate new hypotheses, we did not include any multiple-testing adjustments in our analyses. On the basis of prior findings of 16 loci associated with glucose metabolism, we tested the hypothesis that these genetic variants’ interactions with obesity and lifestyle modifiers influence glucose homeostasis, resulting in altered cancer risk. The small indirect effect could be due to measurement error in the mediators. Since our study was conducted using data from only European-American postmenopausal women, care should be taken when generalizing our findings to other populations.

## Conclusions

Our results suggest that in postmenopausal women, glucose intolerance has a potential role in the risk for breast cancer and CRC. Obesity modulates the glucose metabolism genetic variant–cancer risk association through pathways other than glucose metabolism traits. Further studies are needed to explore these complicated mechanisms. Our study provides insight into gene–lifestyle interactions and suggests data on potential genetic targets for use in clinical trials for cancer prevention and intervention strategies to reduce the cancer risk in postmenopausal women.

## Additional files

Additional file 1: Effect size of glucose metabolism–relevant SNPs on metabolic biomarkers. **Table S1.1.**Effect size of glucose metabolism–relevant SNPs on glucose level in the pathway of glucose metabolism genetic variants, glucose metabolism traits, and breast cancer risk, stratified by obesity status and obesity-related factors. **Table S1.2.** Effect size of glucose metabolism–relevant SNPs on HOMA-IR level in the pathway of glucose metabolism genetic variants, glucose metabolism traits, and breast cancer risk, stratified by obesity status and obesity-related factors. **Table S1.3.** Effect size of glucose metabolism–relevant SNPs on glucose level in the pathway of glucose metabolism genetic variants, glucose metabolism traits, and CRC risk, stratified by obesity status and obesity-related factors. **Table S1.4.** Effect size of glucose metabolism–relevant SNPs on HOMA-IR level in the pathway of glucose metabolism genetic variants, glucose metabolism traits, and CRC risk, stratified by obesity status and obesity-related factors. **Table S1.5.** Effect size of glucose metabolism–relevant SNPs on insulin level in the pathway of glucose metabolism genetic variants, glucose metabolism traits, and breast cancer risk, stratified by obesity status and obesity-related factors. **Table S1.6.** Effect size of glucose metabolism–relevant SNPs on insulin level in the pathway of glucose metabolism genetic variants, glucose metabolism traits, and CRC risk, stratified by obesity status and obesity-related factors. (DOC 188 kb)

Additional file 2: Characteristics of participants. **Table S2.1.** Characteristics of participants, stratified by obesity (measured via waist circumference). **Table S2.2.** Characteristics of participants, stratified by obesity (measured via w/h ratio). **Table S2.3.** Characteristics of participants, stratified by physical activity level. **Table S2.4.** Characteristics of participants, stratified by dietary fat intake. (DOC 387 kb)

Additional file 3: Allele frequencies of 16 glucose metabolism–relevant SNPs. **Table S3.1.** Allele frequencies of 16 glucose metabolism–relevant SNPs, stratified by obesity (measured via BMI). **Table S3.2.** Allele frequencies of 16 glucose metabolism–relevant SNPs, stratified by obesity (measured via waist circumference). **Table S3.3.** Allele frequencies of 16 glucose metabolism–relevant SNPs, stratified by obesity (measured via waist/hip). **Table S3.4.** Allele frequencies of 16 glucose metabolism–relevant SNPs, stratified by physical activity level. **Table S3.5.** Allele frequencies of 16 glucose metabolism–relevant SNPs, stratified by dietary fat intake. (DOC 174 kb)

Additional file 4: Mediation effect of insulin on the relationship between glucose metabolism–relevant SNPs and cancer risk. **Table S4.1.** Mediation effect of insulin on the relationship between glucose metabolism–relevant SNPs and breast cancer risk, stratified by obesity status and obesity-related factors. **Table S4.2.** Mediation effect of insulin on the relationship between glucose metabolism–relevant SNPs and CRC risk, stratified by obesity status and obesity-related factors. (DOC 139 kb)

## Abbreviations

BMI: Body mass index
CI: Confidence interval
CRC: Colorectal cancer
DM: Diabetes mellitus
GWAS: Genome-wide association studies
HOMA-IR: Homeostatic model assessment–insulin resistance
HR: Hazard ratio
IR: Insulin resistance
MET: Metabolic equivalent
SNP: Single-nucleotide polymorphism
w/h: Weight-to-hip ratio
WHI: Women’s health initiative
