# Supplementary material for: Effect of genetic variants and traits related to glucose metabolism and their interaction with obesity on breast and colorectal cancer risk among postmenopausal women
Source: BMC Cancer. 2017 Apr 26;17:290. doi: 10.1186/s12885-017-3284-7 (PMC5405540; doi:10.1186/s12885-017-3284-7)
Supplement: Supplementary file 1 — Effect size of glucose metabolism–relevant SNPs on metabolic biomarkers. Table S1.1.Effect size of glucose metabolism–relevant SNPs on glucose level in the pathway of glucose metabolism genetic variants, glucose metabolism traits, and breast cancer risk, stratified by obesity status and obesity-related factors. Table S1.2. Effect size of glucose metabolism–relevant SNPs on HOMA-IR level in the pathway of glucose metabolism genetic variants, glucose metabolism traits, and breast cancer risk, stratified by obesity status and obesity-related factors. Table S1.3. Effect size of glucose metabolism–relevant SNPs on glucose level in the pathway of glucose metabolism genetic variants, glucose metabolism traits, and CRC risk, stratified by obesity status and obesity-related factors. Table S1.4. Effect size of glucose metabolism–relevant SNPs on HOMA-IR level in the pathway of glucose metabolism genetic variants, glucose metabolism traits, and CRC risk, stratified by obesity status and obesity-related factors. Table S1.5. Effect size of glucose metabolism–relevant SNPs on insulin level in the pathway of glucose metabolism genetic variants, glucose metabolism traits, and breast cancer risk, stratified by obesity status and obesity-related factors. Table S1.6. Effect size of glucose metabolism–relevant SNPs on insulin level in the pathway of glucose metabolism genetic variants, glucose metabolism traits, and CRC risk, stratified by obesity status and obesity-related factors. (DOC 188 kb) [file 12885_2017_3284_MOESM1_ESM.doc]

Table S1.1. Effect size of **glucose metabolism–relevant SNPs** on **glucose level** in the pathway of glucose metabolism genetic variants, glucose metabolism traits, and breast cancer risk, stratified by obesity status and obesity-related factors

|  |  |  | **Non-Obese/**  **Low-Fat Diet Group** | | | |  | **Obese/**  **High-Fat Diet Group** | | | |
| --- | --- | --- | --- | --- | --- | --- | --- | --- | --- | --- | --- |
|  |  |  | **Path a** | | | |  | **Path a** | | | |
| **SNP** | **Nearest gene** | **Effect allele/**  **Other allele** | **Effect size (95% CI)‡ of SNP on *glucose*** | | | |  | **Effect size (95% CI)‡ of SNP on *glucose*** | | | |
|  |  |  | **BMI§** | | | | | | | | |
| rs560887 | *G6PC2* | T/C | **1.45** | **(0.90** | **-** | **2.00)** |  | 0.98 | (-0.54 | - | 2.50) |
|  |  |  | **Waist¶** | | | | | | | | |
| rs560887 | *G6PC2* | T/C | **0.83** | **(0.22** | **-** | **1.43)** |  | **1.71** | **(0.57** | **-** | **2.85)** |
|  |  |  | **waist/hip Ratio€** | | | | | | | | |
| rs560887 | *G6PC2* | T/C | **1.13** | **(0.52** | **-** | **1.74)** |  | **1.43** | **(0.06** | **-** | **2.81)** |
| rs780094 | *GCKR* | C/T | **0.90** | **(0.34** | **-** | **1.46)** |  | 0.72 | (-0.61 | - | 2.05) |
| rs35767 | *IGF1* | A/G | 0.31 | (-0.45 | - | 1.08) |  | -0.01 | (-1.82 | - | 1.80) |
|  |  |  | **Dietary fat intake†** | | | | | | | | |
| rs560887 | *G6PC2* | T/C | **0.97** | **(0.31** | **-** | **1.63)** |  | **2.41** | **(0.99** | **-** | **3.84)** |

BMI, body mass index; CI, confidence interval; SNP, single–nucleotide polymorphism; w/h ratio, waist-to-hip ratio.

Numbers in bold face are statistically significant.

‡ Multiple linear regression was adjusted by covariates (age, education, family history of diabetes mellitus, family history of breast cancer, cardiovascular disease ever, hypertension ever, high cholesterol requiring pills ever, total Healthy Eating Index-2005 score, dietary alcohol and total sugars per day, smoking status, lifetime partner, depressive symptom, oral contraceptive use, history of hysterectomy or oophorectomy, age at menarche, age at menopause, pregnancy history, breastfeeding at least one month, and hormone therapy); effect-modifier variables (physical activity, BMI, and w/h ratio), when not evaluated as effect modifier variables, were adjusted as a covariate; when stratified via waist circumference, w/h ratio was not adjusted.

§ Participants stratified by BMI as non-obese (BMI < 30, n = 3,675) or obese (BMI ≥ 30, n = 1,704).

¶ Participants stratified by waist circumference as non-obese (waist ≤ 88 cm; n = 3,042) or obese (waist > 88 cm; n = 2,337).

€ Participants stratified by w/h as non-obese (w/h ≤ 0.85; n = 3,712) or obese (w/h > 0.85; n = 1,667).

† Participants stratified by dietary fat intake as non-obese (< 40% calories from fat; n = 4,325) or obese (≥ 40% calories from fat; n = 1,054).

Table S1.2. Effect size of **glucose metabolism–relevant SNPs** on **HOMA-IR level** in the pathway of glucose metabolism genetic variants, glucose metabolism traits, and breast cancer risk, stratified by obesity status and obesity-related factors

|  |  |  | **Non-Obese/**  **Low-Fat Diet Group** | | | |  | **Obese/**  **High-Fat Diet Group** | | | |
| --- | --- | --- | --- | --- | --- | --- | --- | --- | --- | --- | --- |
|  |  |  | **Path a** | | | |  | **Path a** | | | |
| **SNP** | **Nearest gene** | **Effect allele/**  **Other allele** | **Effect size (95% CI)‡ of SNP on *HOMA-IR*** | | | |  | **Effect size (95% CI)‡ of SNP on *HOMA-IR*** | | | |
|  |  |  | **BMI§** | | | | | | | | |
| rs560887 | *G6PC2* | T/C | 0.02 | (-0.03 | - | 0.08) |  | -0.01 | (-0.17 | - | 0.15) |
|  |  |  | **Waist¶** | | | | | | | | |
| rs560887 | *G6PC2* | T/C | -0.03 | (-0.08 | - | 0.02) |  | 0.04 | (-0.09 | - | 0.17) |
|  |  |  | **waist/hip Ratio€** | | | | | | | | |
| rs560887 | *G6PC2* | T/C | -0.02 | (-0.07 | - | 0.03) |  | 0.05 | (-0.11 | - | 0.21) |
| rs35767 | *IGF1* | A/G | -0.02 | (-0.09 | - | 0.04) |  | 0.07 | (-0.14 | - | 0.29) |
|  |  |  | **Dietary fat intake†** | | | | | | | | |
| rs560887 | *G6PC2* | T/C | -0.05 | (-0.12 | - | 0.02) |  | **0.21** | **(0.05** | **-** | **0.37)** |

BMI, body mass index; CI, confidence interval; HOMA-IR, homeostatic model assessment–insulin resistance; SNP, single–nucleotide polymorphism; w/h ratio, waist-to-hip ratio. Numbers in bold face are statistically significant.

‡ Multiple linear regression was adjusted by covariates (age, education, family history of diabetes mellitus, family history of breast cancer, cardiovascular disease ever, hypertension ever, high cholesterol requiring pills ever, total Healthy Eating Index-2005 score, dietary alcohol and total sugars per day, smoking status, lifetime partner, depressive symptom, oral contraceptive use, history of hysterectomy or oophorectomy, age at menarche, age at menopause, pregnancy history, breastfeeding at least one month, and hormone therapy); effect-modifier variables (physical activity, BMI, and w/h ratio), when not evaluated as effect modifier variables, were adjusted as a covariate; when stratified via waist circumference, w/h ratio was not adjusted.

§ Participants stratified by BMI as non-obese (BMI < 30, n = 3,675) or obese (BMI ≥ 30, n = 1,704).

¶ Participants stratified by waist circumference as non-obese (waist ≤ 88 cm; n = 3,042) or obese (waist > 88 cm; n = 2,337).

€ Participants stratified by w/h as non-obese (w/h ≤ 0.85; n = 3,712) or obese (w/h > 0.85; n = 1,667).

† Participants stratified by dietary fat intake as non-obese (< 40% calories from fat; n = 4,325) or obese (≥ 40% calories from fat; n = 1,054).

Table S1.3. Effect size of **glucose metabolism–relevant SNPs** on **glucose level** in the pathway of glucose metabolism genetic variants, glucose metabolism traits, and CRC risk, stratified by obesity status and obesity-related factors

|  |  |  | **Non-Obese/Active/**  **Low-Fat Diet Group** | | | |  | **Obese/Inactive/**  **High-Fat Diet Group** | | | |
| --- | --- | --- | --- | --- | --- | --- | --- | --- | --- | --- | --- |
|  |  |  | **Path a** | | | |  | **Path a** | | | |
| **SNP** | **Nearest gene** | **Effect allele/**  **Other allele** | **Effect size (95% CI)‡ of SNP on *glucose*** | | | |  | **Effect size (95% CI)‡ of SNP on *glucose*** | | | |
|  |  |  | **BMI§** | | | | | | | | |
| rs4607517 | *GCK* | G/A | **-1.30** | **(-1.98** | **-** | **-0.62)** |  | -1.41 | (-3.21 | - | 0.39) |
| rs174550 | *FADS1* | T/C | 0.40 | (-0.13 | - | 0.94) |  | -1.26 | (-2.70 | - | 0.17) |
| rs11605924 | *CRY2* | C/A | - 0.20 | (-0.71 | - | 0.30) |  | 0.37 | (-0.99 | - | 1.74) |
|  |  |  | **Waist¶** | | | | | | | | |
| rs560887 | *G6PC2* | T/C | **0.83** | **(0.22** | **-** | **1.43)** |  | **1.67** | **(0.53** | **-** | **2.82)** |
| rs174550 | *FADS1* | T/C | **0.59** | **(0.01** | **-** | **1.18)** |  | -0.90 | (-1.99 | - | 0.18) |
| rs11605924 | *CRY2* | C/A | -0.33 | (-0.89 | - | 0.22) |  | 0.64 | (-0.41 | - | 1.69) |
|  |  |  | **w/h Ratio€** | | | | | | | | |
| rs10885122 | *ADRA2A* | G/T | 0.21 | (-0.64 | - | 1.06) |  | 0.34 | (-1.58 | - | 2.26) |
|  |  |  | **Physical activity level¥** | | | | | | | | |
| rs4607517 | *GCK* | G/A | **-1.61** | **(-2.54** | **-** | **-0.68)** |  | **-1.10** | **(-2.19** | **-** | **-0.02)** |
|  |  |  | **Dietary fat intake†** | | | | | | | | |
| rs4607517 | *GCK* | G/A | **-1.45** | **(-2.26** | **-** | **-0.64)** |  | -0.91 | (-2.68 | - | 0.86) |
| rs11558471 | *SLC30A8* | A/G | **1.37** | **(0.73** | **-** | **2.01)** |  | -0.60 | (-2.02 | - | 0.81) |

BMI, body mass index; CI, confidence interval; CRC, colorectal cancer; HR, hazard ratio; SNP, single–nucleotide polymorphism; w/h ratio, waist-to-hip ratio. Numbers in bold face are statistically significant.

‡ Multiple linear regression was adjusted by covariates (age, education, family history of diabetes mellitus, family history of colorectal cancer, cardiovascular disease ever, hypertension ever, high cholesterol requiring pills ever, total Healthy Eating Index-2005 score, dietary alcohol and total sugars per day, smoking status, lifetime partner, depressive symptom, oral contraceptive use, history of hysterectomy or oophorectomy, age at menarche, age at menopause, pregnancy history, breastfeeding at least one month, and hormone therapy); effect-modifier variables (physical activity, BMI, and w/h ratio), when not evaluated as effect modifier variables, were adjusted as a covariate; when stratified via waist circumference, w/h ratio was not adjusted.

§ Participants stratified by BMI as non-obese (BMI < 30, n = 3,675) or obese (BMI ≥ 30, n = 1,704).

¶ Participants stratified by waist circumference as non-obese (waist ≤ 88 cm; n = 3,042) or obese (waist > 88 cm; n = 2,337).

€ Participants stratified by w/h as non-obese (w/h ≤ 0.85; n = 3,712) or obese (w/h > 0.85; n = 1,667).

¥ Participants stratified by physical activity level as non-obese (MET ≥ 10; n = 2,344) or obese (MET < 10; n = 3,035).

† Participants stratified by dietary fat intake as non-obese (< 40% calories from fat; n = 4,325) or obese (≥ 40% calories from fat; n = 1,054).

Table S1.4. Effect size of **glucose metabolism–relevant SNPs** on **HOMA-IR level** in the pathway of glucose metabolism genetic variants, glucose metabolism traits, and CRC risk, stratified by obesity status and obesity-related factors

|  |  |  | **Non-Obese/Active/**  **Low-Fat Diet Group** | | | |  | **Obese/Inactive/**  **High-Fat Diet Group** | | | |
| --- | --- | --- | --- | --- | --- | --- | --- | --- | --- | --- | --- |
|  |  |  | **Path a** | | | |  | **Path a** | | | |
| **SNP** | **Nearest gene** | **Effect allele/**  **Other allele** | **Effect size (95% CI)‡ of SNP on *HOMA-IR*** | | | |  | **Effect size (95% CI)‡ of SNP on *HOMA-IR*** | | | |
|  |  |  | **BMI§** | | | | | | | | |
| rs2191349 | DGKB/TMEM195 | G/T | 0.02 | (-0.03 | - | 0.07) |  | -0.02 | (-0.17 | - | 0.12) |
| rs4607517 | *GCK* | G/A | -0.05 | (-0.12 | - | 0.01) |  | -0.01 | (-0.20 | - | 0.19) |
| rs174550 | *FADS1* | T/C | -0.01 | (-0.06 | - | 0.05) |  | **-0.18** | **(-0.34** | **-** | **-0.03)** |
| rs11605924 | *CRY2* | C/A | -0.04 | (-0.09 | - | 0.01) |  | 0.01 | (-0.14 | - | 0.16) |
|  |  |  | **Waist¶** | | | | | | | | |
| rs2191349 | DGKB/TMEM195 | G/T | 0.01 | (-0.03 | - | 0.05) |  | 0.03 | (-0.09 | - | 0.15) |
| rs174550 | *FADS1* | T/C | 0.01 | (-0.03 | - | 0.06) |  | -0.12 | (-0.24 | - | 0.004) |
| rs11605924 | *CRY2* | C/A | 0.001 | (-0.04 | - | 0.04) |  | -0.0001 | (-0.12 | - | 0.12) |
|  |  |  | **waist/hip Ratio€** | | | | | | | | |
| rs2191349 | DGKB/TMEM195 | G/T | 0.02 | (-0.02 | - | 0.07) |  | -0.01 | (-0.16 | - | 0.14) |
| rs10885122 | *ADRA2A* | G/T | -0.01 | (-0.08 | - | 0.07) |  | -0.01 | (-0.23 | - | 0.22) |
| rs174550 | *FADS1* | T/C | -0.02 | (-0.06 | - | 0.03) |  | -0.11 | (-0.27 | - | 0.04) |
|  |  |  | **Physical activity level¥** | | | | | | | | |
| rs4607517 | *GCK* | G/A | 0.02 | (-0.07 | - | 0.11) |  | -0.08 | (-0.20 | - | 0.03) |
|  |  |  | **Dietary fat intake†** | | | | | | | | |
| rs4607517 | *GCK* | G/A | -0.05 | (-0.13 | - | 0.03) |  | -0.01 | (-0.21 | - | 0.19) |
| rs11558471 | *SLC30A8* | A/G | 0.05 | (-0.01 | - | 0.12) |  | -0.06 | (-0.22 | - | 0.10) |

BMI, body mass index; CI, confidence interval; CRC, colorectal cancer; HOMA-IR, homeostatic model assessment–insulin resistance; HR, hazard ratio; SNP, single–nucleotide polymorphism; w/h ratio, waist-to-hip ratio. Numbers in bold face are statistically significant.

‡ Multiple linear regression was adjusted by covariates (age, education, family history of diabetes mellitus, family history of colorectal cancer, cardiovascular disease ever, hypertension ever, high cholesterol requiring pills ever, total Healthy Eating Index-2005 score, dietary alcohol and total sugars per day, smoking status, lifetime partner, depressive symptom, oral contraceptive use, history of hysterectomy or oophorectomy, age at menarche, age at menopause, pregnancy history, breastfeeding at least one month, and hormone therapy); effect-modifier variables (physical activity, BMI, and w/h ratio), when not evaluated as effect modifier variables, were adjusted as a covariate; when stratified via waist circumference, w/h ratio was not adjusted.

§ Participants stratified by BMI as non-obese (BMI < 30, n = 3,675) or obese (BMI ≥ 30, n = 1,704).

¶ Participants stratified by waist circumference as non-obese (waist ≤ 88 cm; n = 3,042) or obese (waist > 88 cm; n = 2,337).

€ Participants stratified by w/h as non-obese (w/h ≤ 0.85; n = 3,712) or obese (w/h > 0.85; n = 1,667).

¥ Participants stratified by physical activity level as non-obese (MET ≥ 10; n = 2,344) or obese (MET < 10; n = 3,035).

† Participants stratified by dietary fat intake as non-obese (< 40% calories from fat; n = 4,325) or obese (≥ 40% calories from fat; n = 1,054).

Table S1.5. Effect size of **glucose metabolism–relevant SNPs** on **insulin level** in the pathway of glucose metabolism genetic variants, glucose metabolism traits, and breast cancer risk, stratified by obesity status and obesity-related factors

|  |  |  | **Non-Obese/**  **Low-Fat Diet Group** | | | |  | **Obese/**  **High-Fat Diet Group** | | | |
| --- | --- | --- | --- | --- | --- | --- | --- | --- | --- | --- | --- |
|  |  |  | **Path a** | | | |  | **Path a** | | | |
| **SNP** | **Nearest gene** | **Effect allele/**  **Other allele** | **Effect size (95% CI)‡ of SNP on *insulin*** | | | |  | **Effect size (95% CI)‡ of SNP on *insulin*** | | | |
|  |  |  | **BMI§** | | | | | | | | |
| rs560887 | *G6PC2* | T/C | -0.05 | (-0.27 | - | 0.17) |  | 0.09 | (-0.37 | - | 0.56) |
|  |  |  | **Waist¶** | | | | | | | | |
| rs560887 | *G6PC2* | T/C | **-0.21** | **(-0.40** | **-** | **-0.03)** |  | 0.14 | (-0.27 | - | 0.55) |
|  |  |  | **w/h Ratio€** | | | | | | | | |
| rs560887 | *G6PC2* | T/C | **-0.23** | **(-0.42** | **-** | **-0.04)** |  | 0.33 | (-0.17 | - | 0.83) |
| rs35767 | *IGF1* | A/G | -0.14 | (-0.38 | - | 0.10) |  | 0.31 | (-0.34 | - | 0.97) |
|  |  |  | **Dietary fat intake†** | | | | | | | | |
| rs560887 | *G6PC2* | T/C | -0.21 | (-0.42 | - | 0.004) |  | 0.53 | (-0.04 | - | 1.09) |

BMI, body mass index; CI, confidence interval; SNP, single–nucleotide polymorphism; w/h ratio, waist-to-hip ratio.

Numbers in bold face are statistically significant.

‡ Multiple linear regression was adjusted by covariates (age, education, family history of diabetes mellitus, family history of breast cancer, cardiovascular disease ever, hypertension ever, high cholesterol requiring pills ever, total Healthy Eating Index-2005 score, dietary alcohol and total sugars per day, smoking status, lifetime partner, depressive symptom, oral contraceptive use, history of hysterectomy or oophorectomy, age at menarche, age at menopause, pregnancy history, breastfeeding at least one month, and hormone therapy); effect-modifier variables (physical activity, BMI, and w/h ratio), when not evaluated as effect modifier variables, were adjusted as a covariate; when stratified via waist circumference, w/h ratio was not adjusted.

§ Participants stratified by BMI as non-obese (BMI < 30, n = 3,675) or obese (BMI ≥ 30, n = 1,704).

¶ Participants stratified by waist circumference as non-obese (waist ≤ 88 cm; n = 3,042) or obese (waist > 88 cm; n = 2,337).

€ Participants stratified by w/h as non-obese (w/h ≤ 0.85; n = 3,712) or obese (w/h > 0.85; n = 1,667).

† Participants stratified by dietary fat intake as non-obese (< 40% calories from fat; n = 4,325) or obese (≥ 40% calories from fat; n = 1,054).

Table S1.6. Effect size of **glucose metabolism–relevant SNPs** on **insulin level** in the pathway of glucose metabolism genetic variants, glucose metabolism traits, and CRC risk, stratified by obesity status and obesity-related factors

|  |  |  | **Non-Obese/Active/**  **Low-Fat Diet Group** | | | |  | **Obese/Inactive/**  **High-Fat Diet Group** | | | |
| --- | --- | --- | --- | --- | --- | --- | --- | --- | --- | --- | --- |
|  |  |  | **Path a** | | | |  | **Path a** | | | |
| **SNP** | **Nearest gene** | **Effect allele/**  **Other allele** | **Effect size (95% CI)‡ of SNP on *insulin*** | | | |  | **Effect size (95% CI)‡ of SNP on *insulin*** | | | |
|  |  |  | **BMI§** | | | | | | | | |
| rs4607517 | *GCK* | G/A | -0.09 | (-0.36 | - | 0.18) |  | 0.15 | (-0.40 | - | 0.70) |
| rs174550 | *FADS1* | T/C | -0.03 | (-0.25 | - | 0.18) |  | -0.40 | (-0.83 | - | 0.04) |
| rs11605924 | *CRY2* | C/A | -0.16 | (-0.36 | - | 0.04) |  | -0.09 | (-0.51 | - | 0.32) |
|  |  |  | **Waist¶** | | | | | | | | |
| rs340874 | *PROX1* | C/T | 0.04 | (-0.13 | - | 0.21) |  | 0.01 | (-0.36 | - | 0.38) |
| rs2191349 | *DGKB/TMEM195* | G/T | 0.01 | (-0.16 | - | 0.18) |  | 0.06 | (-0.31 | - | 0.43) |
| rs174550 | *FADS1* | T/C | 0.01 | (-0.17 | - | 0.19) |  | -0.24 | (-0.63 | - | 0.14) |
| rs11605924 | *CRY2* | C/A | 0.04 | (-0.13 | - | 0.21) |  | -0.21 | (-0.58 | - | 0.17) |
|  |  |  | **waist/hip Ratio€** | | | | | | | | |
| rs10885122 | *ADRA2A* | G/T | -0.05 | (-0.32 | - | 0.21) |  | -0.18 | (-0.87 | - | 0.51) |
|  |  |  | **Physical activity level¥** | | | | | | | | |
| rs4607517 | *GCK* | G/A | 0.23 | (-0.11 | - | 0.58) |  | -0.16 | (-0.51 | - | 0.19) |
|  |  |  | **Dietary fat intake†** | | | | | | | | |
| rs4607517 | *GCK* | G/A | -0.02 | (-0.28 | - | 0.24) |  | 0.04 | (-0.66 | - | 0.74) |
| rs11558471 | *SLC30A8* | A/G | 0.04 | (-0.17 | - | 0.24) |  | -0.23 | (-0.79 | - | 0.33) |

BMI, body mass index; CI, confidence interval; CRC, colorectal cancer; SNP, single–nucleotide polymorphism; w/h ratio, waist-to-hip ratio. Numbers in bold face are statistically significant.

‡ Multiple linear regression was adjusted by covariates (age, education, family history of diabetes mellitus, family history of colorectal cancer, cardiovascular disease ever, hypertension ever, high cholesterol requiring pills ever, total Healthy Eating Index-2005 score, dietary alcohol and total sugars per day, smoking status, lifetime partner, depressive symptom, oral contraceptive use, history of hysterectomy or oophorectomy, age at menarche, age at menopause, pregnancy history, breastfeeding at least one month, and hormone therapy); effect-modifier variables (physical activity, BMI, and w/h ratio), when not evaluated as effect modifier variables, were adjusted as a covariate; when stratified via waist circumference, w/h ratio was not adjusted.

§ Participants stratified by BMI as non-obese (BMI < 30, n = 3,675) or obese (BMI ≥ 30, n = 1,704).

¶ Participants stratified by waist circumference as non-obese (waist ≤ 88 cm; n = 3,042) or obese (waist > 88 cm; n = 2,337).

€ Participants stratified by w/h as non-obese (w/h ≤ 0.85; n = 3,712) or obese (w/h > 0.85; n = 1,667).

¥ Participants stratified by physical activity level as non-obese (MET ≥ 10; n = 2,344) or obese (MET < 10; n = 3,035).

† Participants stratified by dietary fat intake as non-obese (< 40% calories from fat; n = 4,325) or obese (≥ 40% calories from fat; n = 1,054).
