# Supplementary material for: Effect of genetic variants and traits related to glucose metabolism and their interaction with obesity on breast and colorectal cancer risk among postmenopausal women
Source: BMC Cancer. 2017 Apr 26;17:290. doi: 10.1186/s12885-017-3284-7 (PMC5405540; doi:10.1186/s12885-017-3284-7)
Supplement: Supplementary file 2 — Characteristics of participants. Table S2.1. Characteristics of participants, stratified by obesity (measured via waist circumference). Table S2.2. Characteristics of participants, stratified by obesity (measured via w/h ratio). Table S2.3. Characteristics of participants, stratified by physical activity level. Table S2.4. Characteristics of participants, stratified by dietary fat intake. (DOC 387 kb) [file 12885_2017_3284_MOESM2_ESM.doc]

Table S2.1. Characteristics of participants, stratified by obesity (measured via waist circumference)

| **Characteristic** | **Non-obese group (waist ≤ 88cm)** | | | |  | **Obese group (waist > 88cm)** | | | |
| --- | --- | --- | --- | --- | --- | --- | --- | --- | --- |
|  | **(n = 3,042)** | | | |  | **(n = 2,337)** | | | |
|  | **n** | **(%)** |  |  |  | **n** | **(%)** |  |  |
| **Age in years, median (range)** | 68 | (50–79) | | |  | 67 | (50–79)* | | |
| **Education** |  |  |  |  |  |  |  |  |  |
| **≤ High school** | 1,031 | (33.9) |  |  |  | 942 | (40.3)* |  |  |
| **> High school** | 2,011 | (66.1) |  |  |  | 1,395 | (59.7) |  |  |
| **Family history of diabetes mellitus** |  |  |  |  |  |  |  |  |  |
| **No** | 2,255 | (74.1) |  |  |  | 1,581 | (67.7)* |  |  |
| **Yes** | 787 | (25.9) |  |  |  | 756 | (32.3) |  |  |
| **Family history of cancer** |  |  |  |  |  |  |  |  |  |
| **No** | 1,092 | (35.9) |  |  |  | 802 | (34.3) |  |  |
| **Yes** | 1,950 | (64.1) |  |  |  | 1,535 | (65.7) |  |  |
| **Family history of breast cancer** |  |  |  |  |  |  |  |  |  |
| **No** | 2,536 | (83.4) |  |  |  | 1,962 | (84.0) |  |  |
| **Yes** | 506 | (16.6) |  |  |  | 375 | (16.0) |  |  |
| **Family history of colorectal cancer** |  |  |  |  |  |  |  |  |  |
| **No** | 2,565 | (84.3) |  |  |  | 1,971 | (84.3) |  |  |
| **Yes** | 477 | (15.7) |  |  |  | 366 | (15.7) |  |  |
| **Cardiovascular disease ever** |  |  |  |  |  |  |  |  |  |
| **No** | 2,635 | (86.6) |  |  |  | 1,956 | (83.7)* |  |  |
| **Yes** | 407 | (13.4) |  |  |  | 381 | (16.3) |  |  |
| **Hypertension ever** |  |  |  |  |  |  |  |  |  |
| **No** | 2,315 | (76.1) |  |  |  | 1,413 | (60.5)* |  |  |
| **Yes** | 727 | (23.9) |  |  |  | 924 | (39.5) |  |  |
| **High cholesterol requiring pills ever** |  |  |  |  |  |  |  |  |  |
| **No** | 2,677 | (88.0) |  |  |  | 1,961 | (83.9)* |  |  |
| **Yes** | 365 | (12.0) |  |  |  | 376 | (16.1) |  |  |
| **Smoking status** |  |  |  |  |  |  |  |  |  |
| **Never** | 1,609 | (52.9) |  |  |  | 1,170 | (50.1) |  |  |
| **Past** | 1,202 | (39.5) |  |  |  | 1,001 | (42.8) |  |  |
| **Current** | 231 | (7.6) |  |  |  | 166 | (7.1) |  |  |
| **Lifetime partner** |  |  |  |  |  |  |  |  |  |
| **Have never had sex** | 43 | (1.4) |  |  |  | 51 | (2.2)* |  |  |
| **Have had sex** | 2,999 | (98.6) |  |  |  | 2,286 | (97.8) |  |  |
| **Depressive symptom†** |  |  |  |  |  |  |  |  |  |
| **< 0.06** | 2,812 | (92.4) |  |  |  | 2,136 | (91.4) |  |  |
| **≥ 0.06** | 230 | (7.6) |  |  |  | 201 | (8.6) |  |  |
| **METs·hour·week^-1^¶** |  |  |  |  |  |  |  |  |  |
| **< 10** | 1,452 | (47.7) |  |  |  | 1,583 | (67.7)* |  |  |
| **≥ 10** | 1,590 | (52.3) |  |  |  | 754 | (32.3) |  |  |
| **Total HEI**-**2005 score, median (range)‡** | 69.5 | (25.8–90.8) | | |  | 65.6 | (27.9–91.2)* | | |
| **Dietary total sugars in g, median (range)** | 92.4 | (4.6–342.2) | | |  | 92.5 | (10.0–474.5) | | |
| **Dietary alcohol per day in g, median (range)** | 1.559 | (0.0–106.4) | | |  | 0.923 | (0.0–148.6)* | | |
| **% calories from fat¥** |  |  | | |  |  |  | | |
| **< 40%** | 2,562 | (84.2) | | |  | 1,763 | (75.4)* | | |
| **≥ 40%** | 480 | (15.8) | | |  | 574 | (24.6) | | |

Table S2.1 (Continued)

| **Characteristic** | **Non-obese group (waist ≤ 88cm)** | | | |  | **Obese group (waist > 88cm)** | | | |
| --- | --- | --- | --- | --- | --- | --- | --- | --- | --- |
|  | **(n = 3,042)** | | | |  | **(n = 2,337)** | | | |
|  | **n** | **(%)** |  |  |  | **n** | **(%)** |  |  |
| **BMI, kg/m^2^, median (range)** | 24.6 | (13.8–69.4) | | |  | 31.6 | (20.1–62.0)* | | |
| **Oral contraceptive use** |  |  |  |  |  |  |  |  |  |
| **Never** | 2,021 | (66.4) |  |  |  | 1,550 | (66.3) |  |  |
| **Ever** | 1,021 | (33.6) |  |  |  | 787 | (33.7) |  |  |
| **History of hysterectomy or oophorectomy** |  |  |  |  |  |  |  |  |  |
| **No** | 2,071 | (68.1) |  |  |  | 1,392 | (59.6)* |  |  |
| **Yes** | 971 | (31.9) |  |  |  | 945 | (40.4) |  |  |
| **Age at menarche in years, median (range)** | 13 | (≤ 9–≥ 17) | | |  | 13 | (≤ 9–≥ 17) | | |
| **Age at menopause in years, median (range)** | 50 | (21–60) | | |  | 50 | (20–60) | | |
| **Pregnancy history** |  |  |  |  |  |  |  |  |  |
| **No** | 234 | (7.7) |  |  |  | 176 | (7.5) |  |  |
| **Yes** | 2,808 | (92.3) |  |  |  | 2,161 | (92.5) |  |  |
| **Breastfeeding at least one month** |  |  |  |  |  |  |  |  |  |
| **No** | 1,347 | (44.3) |  |  |  | 1,114 | (47.7)* |  |  |
| **Yes** | 1,695 | (55.7) |  |  |  | 1,223 | (52.3) |  |  |
| **Exogenous estrogen use** |  |  |  |  |  |  |  |  |  |
| **No** | 1,848 | (60.7) |  |  |  | 1,498 | (64.1)* |  |  |
| **Yes** | 1,194 | (39.3) |  |  |  | 839 | (35.9) |  |  |
| **Glucose in mg/dl, median (range)** | 92.0 | (50.0–369.0) | | |  | 96.0 | (39.0–347.0)* | | |
| **Insulin in μIU/ml, median (range)** | 5.2 | (0.5–30.0) | | |  | 9.2 | (0.3–119.4)* | | |
| **HOMA-IR, median (range)** | 1.2 | (0.1–7.5) | | |  | 2.2 | (0.1–42.3)* | | |

BMI, body mass index; HEI-2005, Healthy Eating Index-2005; HOMA-IR, homeostatic model assessment–insulin resistance; MET, metabolic equivalent.

* *p* < 0.05, chi-squared or Wilcoxon’s rank-sum test.

**†** Depression scales were estimated by using a short form of the Center for Epidemiologic Studies Depression Scale and categorized with 0.06 as the cutoff to detect depressive disorders.

¶ Physical activity was estimated from recreational physical activity combining walking and mild, moderate, and strenuous physical activity.

‡ HEI-2005 is a measure of diet quality that assesses adherence to the U.S. Department of Agriculture’s Dietary Guidelines for Americans. The total HEI score ranges from 0 to 100, with higher scores indicating higher diet quality.

¥ Participants were stratified by high-fat diet using 40% as a cutoff value relevant to glucose intolerance[47].

Table S2.2. Characteristics of participants, stratified by obesity (measured via w/h ratio)

| **Characteristic** | **Non-obese group (w/h ≤ 0.85)** | | | |  | **Obese group (w/h > 0.85)** | | | |
| --- | --- | --- | --- | --- | --- | --- | --- | --- | --- |
|  | **(n = 3,712)** | | | |  | **(n = 1,667)** | | | |
|  | **n** | **(%)** |  |  |  | **n** | **(%)** |  |  |
| **Age in years, median (range)** | 68 | (50–79) | | |  | 68 | (50–79) | | |
| **Education** |  |  |  |  |  |  |  |  |  |
| **≤ High school** | 1,305 | (35.2) |  |  |  | 668 | (40.1)* |  |  |
| **> High school** | 2,407 | (64.8) |  |  |  | 999 | (59.9) |  |  |
| **Family history of diabetes mellitus** |  |  |  |  |  |  |  |  |  |
| **No** | 2,696 | (72.6) |  |  |  | 1,140 | (68.4)* |  |  |
| **Yes** | 1,016 | (27.4) |  |  |  | 527 | (31.6) |  |  |
| **Family history of cancer** |  |  |  |  |  |  |  |  |  |
| **No** | 1,312 | (35.3) |  |  |  | 582 | (34.9) |  |  |
| **Yes** | 2,400 | (64.7) |  |  |  | 1,085 | (65.1) |  |  |
| **Family history of breast cancer** |  |  |  |  |  |  |  |  |  |
| **No** | 3,108 | (83.7) |  |  |  | 1,390 | (83.4) |  |  |
| **Yes** | 604 | (16.3) |  |  |  | 277 | (16.6) |  |  |
| **Family history of colorectal cancer** |  |  |  |  |  |  |  |  |  |
| **No** | 3,137 | (84.5) |  |  |  | 1,399 | (83.9) |  |  |
| **Yes** | 575 | (15.5) |  |  |  | 268 | (16.1) |  |  |
| **Cardiovascular disease ever** |  |  |  |  |  |  |  |  |  |
| **No** | 3,190 | (85.9) |  |  |  | 1,401 | (84.0) |  |  |
| **Yes** | 522 | (14.1) |  |  |  | 266 | (16.0) |  |  |
| **Hypertension ever** |  |  |  |  |  |  |  |  |  |
| **No** | 2,738 | (73.8) |  |  |  | 990 | (59.4)* |  |  |
| **Yes** | 974 | (26.2) |  |  |  | 677 | (40.6) |  |  |
| **High cholesterol requiring pills ever** |  |  |  |  |  |  |  |  |  |
| **No** | 3,278 | (88.3) |  |  |  | 1,360 | (81.6)* |  |  |
| **Yes** | 434 | (11.7) |  |  |  | 307 | (18.4) |  |  |
| **Smoking status** |  |  |  |  |  |  |  |  |  |
| **Never** | 2,010 | (54.1) |  |  |  | 769 | (46.1)* |  |  |
| **Past** | 1,459 | (39.3) |  |  |  | 744 | (44.6) |  |  |
| **Current** | 243 | (6.5) |  |  |  | 154 | (9.2) |  |  |
| **Lifetime partner** |  |  |  |  |  |  |  |  |  |
| **Have never had sex** | 56 | (1.5) |  |  |  | 38 | (2.3) |  |  |
| **Have had sex** | 3,656 | (98.5) |  |  |  | 1,629 | (97.7) |  |  |
| **Depressive symptom†** |  |  |  |  |  |  |  |  |  |
| **< 0.06** | 3,426 | (92.3) |  |  |  | 1,522 | (91.3) |  |  |
| **≥ 0.06** | 286 | (7.7) |  |  |  | 145 | (8.7) |  |  |
| **METs·hour·week^-1^¶** |  |  |  |  |  |  |  |  |  |
| **< 10** | 1,974 | (53.2) |  |  |  | 1,061 | (63.6)* |  |  |
| **≥ 10** | 1,738 | (46.8) |  |  |  | 606 | (36.4) |  |  |
| **Total HEI**-**2005 score, median (range)‡** | 68.7 | (25.8–90.8) | | |  | 65.5 | (27.9–91.2)* | | |
| **Dietary total sugars in g, median (range)** | 92.1 | (4.6–441.6) | | |  | 93.4 | (10.0–474.5) | | |
| **Dietary alcohol per day in g, median (range)** | 1.036 | (0.0–106.7) | | |  | 1.009 | (0.0–148.6) | | |
| **% calories from fat¥** |  |  | | |  |  |  | | |
| **< 40%** | 3,043 | (82.0) | | |  | 1,282 | (76.9)* | | |
| **≥ 40%** | 669 | (18.0) | | |  | 385 | (23.1) | | |
| **BMI in kg/m^2^, median (range)** | 26.1 | (13.8–69.4) | | |  | 30.0 | (18.3–56.9)* | | |

Table S2.2 (Continued)

| **Characteristic** | **Non-obese group (w/h ≤ 0.85)** | | | |  | **Obese group (w/h > 0.85)** | | | |
| --- | --- | --- | --- | --- | --- | --- | --- | --- | --- |
|  | **(n = 3,712)** | | | |  | **(n = 1,667)** | | | |
|  | **n** | **(%)** |  |  |  | **n** | **(%)** |  |  |
| **Oral contraceptive use** |  |  |  |  |  |  |  |  |  |
| **Never** | 2,448 | (65.9) |  |  |  | 1,123 | (67.4) |  |  |
| **Ever** | 1,264 | (34.1) |  |  |  | 544 | (32.6) |  |  |
| **History of hysterectomy or oophorectomy** |  |  |  |  |  |  |  |  |  |
| **No** | 2,458 | (66.2) |  |  |  | 1,005 | (60.3)* |  |  |
| **Yes** | 1,254 | (33.8) |  |  |  | 662 | (39.7) |  |  |
| **Age at menarche in years, median (range)** | 13 | (≤ 9–≥ 17) | | |  | 13 | (≤ 9–≥ 17) | | |
| **Age at menopause in years, median (range)** | 50 | (21–60) | | |  | 50 | (20–60) | | |
| **Pregnancy history** |  |  |  |  |  |  |  |  |  |
| **No** | 278 | (7.5) |  |  |  | 132 | (7.9) |  |  |
| **Yes** | 3,434 | (92.5) |  |  |  | 1,535 | (92.1) |  |  |
| **Breastfeeding at least one month** |  |  |  |  |  |  |  |  |  |
| **No** | 1,660 | (44.7) |  |  |  | 801 | (48.1)* |  |  |
| **Yes** | 2,052 | (55.3) |  |  |  | 866 | (51.9) |  |  |
| **Exogenous estrogen use** |  |  |  |  |  |  |  |  |  |
| **No** | 2,281 | (61.4) |  |  |  | 1,065 | (63.9) |  |  |
| **Yes** | 1,431 | (38.6) |  |  |  | 602 | (36.1) |  |  |
| **Glucose in mg/dl, median (range)** | 92.0 | (39.0–369.0) | | |  | 96.0 | (62.0–320.0)* | | |
| **Insulin in μIU/ml, median (range)** | 5.8 | (0.3–47.8) | | |  | 8.9 | (1.4–119.4)* | | |
| **HOMA-IR, median (range)** | 1.3 | (0.1–12.1) | | |  | 2.1 | (0.3–42.3)* | | |

BMI, body mass index; HEI-2005, Healthy Eating Index-2005; HOMA-IR, homeostatic model assessment–insulin resistance; MET, metabolic equivalent; w/h, waist-to-hip ratio.

* *p* < 0.05, chi-squared or Wilcoxon’s rank-sum test.

**†** Depression scales were estimated by using a short form of the Center for Epidemiologic Studies Depression Scale and categorized with 0.06 as the cutoff to detect depressive disorders.

¶ Physical activity was estimated from recreational physical activity combining walking and mild, moderate, and strenuous physical activity.

‡ HEI-2005 is a measure of diet quality that assesses adherence to the U.S. Department of Agriculture’s Dietary Guidelines for Americans. The total HEI score ranges from 0 to 100, with higher scores indicating higher diet quality.

¥ Participants were stratified by high-fat diet using 40% as a cutoff value relevant to glucose intolerance[47].

Table S2.3. Characteristics of participants, stratified by physical activity level

| **Characteristic** | **High physical activity group**  **(MET ≥ 10)** | | | |  | **Low physical activity group**  **(MET < 10)** | | | |
| --- | --- | --- | --- | --- | --- | --- | --- | --- | --- |
|  | **(n = 2,344)** | | | |  | **(n = 3,035)** | | | |
|  | **n** | **(%)** |  |  |  | **n** | **(%)** |  |  |
| **Age in years, median (range)** | 68 | (50–79) | | |  | 67 | (50–79)* | | |
| **Education** |  |  |  |  |  |  |  |  |  |
| **≤ High school** | 705 | (30.1) |  |  |  | 1,268 | (41.8)* |  |  |
| **> High school** | 1,639 | (69.9) |  |  |  | 1,767 | (58.2) |  |  |
| **Family history of diabetes mellitus** |  |  |  |  |  |  |  |  |  |
| **No** | 1,676 | (71.5) |  |  |  | 2,160 | (71.2) |  |  |
| **Yes** | 668 | (28.5) |  |  |  | 875 | (28.8) |  |  |
| **Family history of cancer** |  |  |  |  |  |  |  |  |  |
| **No** | 832 | (35.5) |  |  |  | 1,062 | (35.0) |  |  |
| **Yes** | 1,512 | (64.5) |  |  |  | 1,973 | (65.0) |  |  |
| **Family history of breast cancer** |  |  |  |  |  |  |  |  |  |
| **No** | 1,935 | (82.6) |  |  |  | 2,563 | (84.4) |  |  |
| **Yes** | 409 | (17.4) |  |  |  | 472 | (15.6) |  |  |
| **Family history of colorectal cancer** |  |  |  |  |  |  |  |  |  |
| **No** | 1,955 | (83.4) |  |  |  | 2,581 | (85.0) |  |  |
| **Yes** | 389 | (16.6) |  |  |  | 454 | (15.0) |  |  |
| **Cardiovascular disease ever** |  |  |  |  |  |  |  |  |  |
| **No** | 2,039 | (87.0) |  |  |  | 2,552 | (84.1)* |  |  |
| **Yes** | 305 | (13.0) |  |  |  | 483 | (15.9) |  |  |
| **Hypertension ever** |  |  |  |  |  |  |  |  |  |
| **No** | 1,677 | (71.5) |  |  |  | 2,051 | (67.6)* |  |  |
| **Yes** | 667 | (28.5) |  |  |  | 984 | (32.4) |  |  |
| **High cholesterol requiring pills ever** |  |  |  |  |  |  |  |  |  |
| **No** | 2,034 | (86.8) |  |  |  | 2,604 | (85.8) |  |  |
| **Yes** | 310 | (13.2) |  |  |  | 431 | (14.2) |  |  |
| **Smoking status** |  |  |  |  |  |  |  |  |  |
| **Never** | 1,181 | (50.4) |  |  |  | 1,598 | (52.7)* |  |  |
| **Past** | 1,046 | (44.6) |  |  |  | 1,157 | (38.1) |  |  |
| **Current** | 117 | (5.0) |  |  |  | 280 | (9.2) |  |  |
| **Lifetime partner** |  |  |  |  |  |  |  |  |  |
| **Have never had sex** | 40 | (1.7) |  |  |  | 54 | (1.8) |  |  |
| **Have had sex** | 2,304 | (98.3) |  |  |  | 2,981 | (98.2) |  |  |
| **Depressive symptom†** |  |  |  |  |  |  |  |  |  |
| **< 0.06** | 2,203 | (94.0) |  |  |  | 2,745 | (90.4)* |  |  |
| **≥ 0.06** | 141 | (6.0) |  |  |  | 290 | (9.6) |  |  |
| **Total HEI**-**2005 score, median (range)‡** | 70.7 | (28.2–90.8) | | |  | 65.4 | (25.8–91.2)* | | |
| **Dietary total sugars in g, median (range)** | 95.8 | (15.0–342.2) | | |  | 89.8 | (4.6–474.5)* | | |
| **Dietary alcohol per day in g, median (range)** | 1.852 | (0.0–148.6) | | |  | 0.846 | (0.0–148.1)* | | |
| **% calories from fat¥** |  |  | | |  |  |  | | |
| **< 40%** | 2,056 | (87.7) | | |  | 2,269 | (74.8)* | | |
| **≥ 40%** | 288 | (12.3) | | |  | 766 | (25.2) | | |
| **BMI in kg/m^2^, median (range)** | 26.1 | (17.2–69.4) | | |  | 28.4 | (13.8–66.1)* | | |
| **Waist circumference in cm, median (range)** | 82.5 | (37.5–190.0) | | |  | 89.0 | (56.0–191.8)* | | |
| **Waist-to-hip ratio, median (range)** | 0.801 | (0.341–1.893) | | |  | 0.821 | (0.498–1.828)* | | |

Table S2.3 (Continued)

| **Characteristic** | **High physical activity group**  **(MET ≥ 10)** | | | |  | **Low physical activity group**  **(MET < 10)** | | | |
| --- | --- | --- | --- | --- | --- | --- | --- | --- | --- |
|  | **(n = 2,344)** | | | |  | **(n = 3,035)** | | | |
|  | **n** | **(%)** |  |  |  | **n** | **(%)** |  |  |
| **Oral contraceptive use** |  |  |  |  |  |  |  |  |  |
| **Never** | 1,542 | (65.8) |  |  |  | 2,029 | (66.9) |  |  |
| **Ever** | 802 | (34.2) |  |  |  | 1,006 | (33.1) |  |  |
| **History of hysterectomy or oophorectomy** |  |  |  |  |  |  |  |  |  |
| **No** | 1,580 | (67.4) |  |  |  | 1,883 | (62.0)* |  |  |
| **Yes** | 764 | (32.6) |  |  |  | 1,152 | (38.0) |  |  |
| **Age at menarche in years, median (range)** | 13 | (≤ 9–≥ 17) | | |  | 13 | (≤ 9–≥ 17) | | |
| **Age at menopause in years, median (range)** | 50 | (26–60) | | |  | 50 | (20–60) | | |
| **Pregnancy history** |  |  |  |  |  |  |  |  |  |
| **No** | 168 | (7.2) |  |  |  | 242 | (8.0) |  |  |
| **Yes** | 2,176 | (92.8) |  |  |  | 2,793 | (92.0) |  |  |
| **Breastfeeding at least one month** |  |  |  |  |  |  |  |  |  |
| **No** | 1,049 | (44.8) |  |  |  | 1,412 | (46.5) |  |  |
| **Yes** | 1,295 | (55.2) |  |  |  | 1,623 | (53.5) |  |  |
| **Exogenous estrogen use** |  |  |  |  |  |  |  |  |  |
| **No** | 1,397 | (59.6) |  |  |  | 1,949 | (64.2)* |  |  |
| **Yes** | 947 | (40.4) |  |  |  | 1,086 | (35.8) |  |  |
| **Glucose in mg/dl, median (range)** | 92.0 | (39.0–369.0) | | |  | 94.0 | (50.0–347.0)* | | |
| **Insulin in μIU/ml, median (range)** | 5.8 | (0.5–119.4) | | |  | 7.2 | (0.3–104.1)* | | |
| **HOMA-IR, median (range)** | 1.3 | (0.1–25.1) | | |  | 1.7 | (0.1–42.3)* | | |

BMI, body mass index; HEI-2005, Healthy Eating Index-2005; HOMA-IR, homeostatic model assessment–insulin resistance; MET, metabolic equivalent.

* *p* < 0.05, chi-squared or Wilcoxon’s rank-sum test.

**†** Depression scales were estimated by using a short form of the Center for Epidemiologic Studies Depression Scale and categorized with 0.06 as the cutoff to detect depressive disorders.

¶ Physical activity was estimated from recreational physical activity combining walking and mild, moderate, and strenuous physical activity.

‡ HEI-2005 is a measure of diet quality that assesses adherence to the U.S. Department of Agriculture’s Dietary Guidelines for Americans. The total HEI score ranges from 0 to 100, with higher scores indicating higher diet quality.

¥ Participants were stratified by high-fat diet using 40% as a cutoff value relevant to glucose intolerance[47].

Table S2.4. Characteristics of participants, stratified by dietary fat intake

| **Characteristic** | **Low dietary fat intake group**  **(< 40.0% calories from fat)** | | | |  | **High dietary fat intake group**  **(≥ 40.0% calories from fat)** | | | |
| --- | --- | --- | --- | --- | --- | --- | --- | --- | --- |
|  | **(n = 4,325)** | | | |  | **(n = 1,054)** | | | |
|  | **n** | **(%)** |  |  |  | **n** | **(%)** |  |  |
| **Age in years, median (range)** | 68 | (50–79) | | |  | 67 | (50–79)* | | |
| **Education** |  |  |  |  |  |  |  |  |  |
| **≤ High school** | 1,489 | (34.4) |  |  |  | 484 | (45.9)* |  |  |
| **> High school** | 2,836 | (65.6) |  |  |  | 570 | (54.1) |  |  |
| **Family history of diabetes mellitus** |  |  |  |  |  |  |  |  |  |
| **No** | 3,084 | (71.3) |  |  |  | 752 | (71.3) |  |  |
| **Yes** | 1,241 | (28.7) |  |  |  | 302 | (28.7) |  |  |
| **Family history of cancer** |  |  |  |  |  |  |  |  |  |
| **No** | 1,522 | (35.2) |  |  |  | 372 | (35.3) |  |  |
| **Yes** | 2,803 | (64.8) |  |  |  | 682 | (64.7) |  |  |
| **Family history of breast cancer** |  |  |  |  |  |  |  |  |  |
| **No** | 3,631 | (84.0) |  |  |  | 867 | (82.3) |  |  |
| **Yes** | 694 | (16.0) |  |  |  | 187 | (17.7) |  |  |
| **Family history of colorectal cancer** |  |  |  |  |  |  |  |  |  |
| **No** | 3,631 | (84.0) |  |  |  | 905 | (85.9) |  |  |
| **Yes** | 694 | (16.0) |  |  |  | 149 | (14.1) |  |  |
| **Cardiovascular disease ever** |  |  |  |  |  |  |  |  |  |
| **No** | 3,690 | (85.3) |  |  |  | 901 | (85.5) |  |  |
| **Yes** | 635 | (14.7) |  |  |  | 153 | (14.5) |  |  |
| **Hypertension ever** |  |  |  |  |  |  |  |  |  |
| **No** | 2,984 | (69.0) |  |  |  | 744 | (70.6) |  |  |
| **Yes** | 1,341 | (31.0) |  |  |  | 310 | (29.4) |  |  |
| **High cholesterol requiring pills ever** |  |  |  |  |  |  |  |  |  |
| **No** | 3,693 | (85.4) |  |  |  | 945 | (89.7)* |  |  |
| **Yes** | 632 | (14.6) |  |  |  | 109 | (10.3) |  |  |
| **Smoking status** |  |  |  |  |  |  |  |  |  |
| **Never** | 2,239 | (51.8) |  |  |  | 540 | (51.2)* |  |  |
| **Past** | 1,818 | (42.0) |  |  |  | 385 | (36.5) |  |  |
| **Current** | 268 | (6.2) |  |  |  | 129 | (12.2) |  |  |
| **Lifetime partner** |  |  |  |  |  |  |  |  |  |
| **Have never had sex** | 81 | (1.9) |  |  |  | 13 | (1.2) |  |  |
| **Have had sex** | 4,244 | (98.1) |  |  |  | 1,041 | (98.8) |  |  |
| **Depressive symptom†** |  |  |  |  |  |  |  |  |  |
| **< 0.06** | 4,013 | (92.8) |  |  |  | 935 | (88.7)* |  |  |
| **≥ 0.06** | 312 | (7.2) |  |  |  | 119 | (11.3) |  |  |
| **METs·hour·week^-1^¶** |  |  |  |  |  |  |  |  |  |
| **< 10** | 2,269 | (52.5) |  |  |  | 766 | (72.7)* |  |  |
| **≥ 10** | 2,056 | (47.5) |  |  |  | 288 | (27.3) |  |  |
| **Total HEI**-**2005 score, median (range)‡** | 70.4 | (27.9–91.2) | | |  | 57.4 | (25.8–84.2)* | | |
| **Dietary total sugars in g, median (range)** | 95.5 | (10.0–474.5) | | |  | 79.0 | (4.6–350.4)* | | |
| **Dietary alcohol per day in g, median (range)** | 1.174 | (0.0–148.6) | | |  | 0.605 | (0.0–57.6)* | | |
| **BMI in kg/m^2^, median (range)** | 27.0 | (13.8–69.4) | | |  | 28.8 | (17.2–66.1)* | | |
| **Waist circumference in cm, median (range)** | 85.0 | (54.5–191.8) | | |  | 90.0 | (37.5–144.5)* | | |
| **Waist-to-hip ratio, median (range)** | 0.809 | (0.341–1.893) | | |  | 0.825 | (0.364–1.280)* | | |

Table S2.4 (Continued)

| **Characteristic** | **Low dietary fat intake group**  **(< 40.0% calories from fat)** | | | |  | **High dietary fat intake group**  **(≥ 40.0% calories from fat)** | | | |
| --- | --- | --- | --- | --- | --- | --- | --- | --- | --- |
|  | **(n = 4,325)** | | | |  | **(n = 1,054)** | | | |
|  | **n** | **(%)** |  |  |  | **n** | **(%)** |  |  |
| **Oral contraceptive use** |  |  |  |  |  |  |  |  |  |
| **Never** | 2,869 | (66.3) |  |  |  | 702 | (66.6) |  |  |
| **Ever** | 1,456 | (33.7) |  |  |  | 352 | (33.4) |  |  |
| **History of hysterectomy or oophorectomy** |  |  |  |  |  |  |  |  |  |
| **No** | 2,815 | (65.1) |  |  |  | 648 | (61.5)* |  |  |
| **Yes** | 1,510 | (34.9) |  |  |  | 406 | (38.5) |  |  |
| **Age at menarche in years, median (range)** | 13 | (≤ 9–≥ 17) | | |  | 13 | (≤ 9–≥ 17) | | |
| **Age at menopause in years, median (range)** | 50 | (21–60) | | |  | 49 | (20–60)* | | |
| **Pregnancy history** |  |  |  |  |  |  |  |  |  |
| **No** | 331 | (7.7) |  |  |  | 79 | (7.5) |  |  |
| **Yes** | 3,994 | (92.3) |  |  |  | 975 | (92.5) |  |  |
| **Breastfeeding at least one month** |  |  |  |  |  |  |  |  |  |
| **No** | 1,959 | (45.3) |  |  |  | 502 | (47.6) |  |  |
| **Yes** | 2,366 | (54.7) |  |  |  | 552 | (52.4) |  |  |
| **Exogenous estrogen use** |  |  |  |  |  |  |  |  |  |
| **No** | 2,674 | (61.8) |  |  |  | 672 | (63.8) |  |  |
| **Yes** | 1,651 | (38.2) |  |  |  | 382 | (36.2) |  |  |
| **Glucose in mg/dl, median (range)** | 93.0 | (39.0–369.0) | | |  | 95.0 | (67.0–309.0)* | | |
| **Insulin in μIU/ml, median (range)** | 6.3 | (0.3–119.4) | | |  | 7.9 | (0.5–104.1)* | | |
| **HOMA-IR, median (range)** | 1.5 | (0.1–42.3) | | |  | 1.9 | (0.1–24.7)* | | |

BMI, body mass index; HEI-2005, Healthy Eating Index-2005; HOMA-IR, homeostatic model assessment–insulin resistance; MET, metabolic equivalent.

* *p* < 0.05, chi-squared or Wilcoxon’s rank-sum test.

**†** Depression scales were estimated by using a short form of the Center for Epidemiologic Studies Depression Scale and categorized with 0.06 as the cutoff to detect depressive disorders.

¶ Physical activity was estimated from recreational physical activity combining walking and mild, moderate, and strenuous physical activity.

‡ HEI-2005 is a measure of diet quality that assesses adherence to the U.S. Department of Agriculture’s Dietary Guidelines for Americans. The total HEI score ranges from 0 to 100, with higher scores indicating higher diet quality.

¥ Participants were stratified by high-fat diet using 40% as a cutoff value relevant to glucose intolerance[47].
