# Supplementary material for: Effect of genetic variants and traits related to glucose metabolism and their interaction with obesity on breast and colorectal cancer risk among postmenopausal women
Source: BMC Cancer. 2017 Apr 26;17:290. doi: 10.1186/s12885-017-3284-7 (PMC5405540; doi:10.1186/s12885-017-3284-7)
Supplement: Supplementary file 3 — Allele frequencies of 16 glucose metabolism–relevant SNPs. Table S3.1. Allele frequencies of 16 glucose metabolism–relevant SNPs, stratified by obesity (measured via BMI). Table S3.2. Allele frequencies of 16 glucose metabolism–relevant SNPs, stratified by obesity (measured via waist circumference). Table S3.3. Allele frequencies of 16 glucose metabolism–relevant SNPs, stratified by obesity (measured via waist/hip). Table S3.4. Allele frequencies of 16 glucose metabolism–relevant SNPs, stratified by physical activity level. Table S3.5. Allele frequencies of 16 glucose metabolism–relevant SNPs, stratified by dietary fat intake. (DOC 174 kb) [file 12885_2017_3284_MOESM3_ESM.doc]

Table S3.1. Allele frequencies of 16 glucose metabolism–relevant SNPs, stratified by obesity (measured via BMI)

| **SNP** | **Chromosome** | **Nearest gene** | **Allele**  **(effect/baseline)** | **Effect allele frequency** | | |
| --- | --- | --- | --- | --- | --- | --- |
|  |  |  |  | **Non-obese group**  **(BMI < 30.0)** |  | **Obese group**  **(BMI ≥ 30.0)** |
|  |  |  |  | **(n = 3,675)** |  | **(n = 1,704)** |
| **rs340874** | 1 | *PROX1* | C/T | 55.4 |  | 55.2 |
| **rs560887** | 2 | *G6PC2* | T/C | 29.4 |  | 30.0 |
| **rs780094** | 2 | *GCKR* | C/T | 60.1 |  | 59.9 |
| **rs11708067** | 3 | *ADCY5* | A/G | 76.9 |  | 76.9 |
| **rs11920090** | 3 | *SLC2A2* | T/A | 86.3 |  | 87.5 |
| **rS3191349** | 7 | *DGKB/TMEM195* | G/T | 45.7 |  | 44.1 |
| **rs4607517** | 7 | *GCK* | G/A | 83.1 |  | 82.4 |
| **rs11558471** | 8 | *SLC30A8* | A/G | 68.9 |  | 66.8 |
| **rs7034200** | 9 | *GLIS3* | A/C | 49.4 |  | 50.2 |
| **rs4506565** | 10 | *TCF7L2* | A/T | 68.6 |  | 69.8 |
| **rs10885122** | 10 | *ADRA2A* | G/T | 88.0 |  | 87.9 |
| **rs174550** | 11 | *FADS1* | T/C | 65.7 |  | 65.5 |
| **rs7944584** | 11 | *MADD* | A/T | 72.4 |  | 73.0 |
| **rs11605924** | 11 | *CRY2* | C/A | 52.0 |  | 53.4 |
| **rs11071657** | 15 | *C2CD4B* | A/G | 62.9 |  | 62.2 |
| **rs35767** | 12 | *IGF1* | A/G | 15.1 |  | 14.5 |

BMI, body mass index; SNP, single-nucleotide polymorphism

Table S3.2. Allele frequencies of 16 glucose metabolism–relevant SNPs, stratified by obesity (measured via waist circumference)

| **SNP** | **Chromosome** | **Nearest gene** | **Allele**  **(effect/baseline)** | **Effect allele frequency** | | |
| --- | --- | --- | --- | --- | --- | --- |
|  |  |  |  | **Non-obese group**  **(Waist ≤ 88 cm)** |  | **Obese group**  **(Waist > 88 cm)** |
|  |  |  |  | **(n = 3,042)** |  | **(n = 2,337)** |
| **rs340874** | 1 | *PROX1* | C/T | 55.8 |  | 54.7 |
| **rs560887** | 2 | *G6PC2* | T/C | 29.2 |  | 30.0 |
| **rs780094** | 2 | *GCKR* | C/T | 60.7 |  | 59.2 |
| **rs11708067** | 3 | *ADCY5* | A/G | 76.7 |  | 77.2 |
| **rs11920090** | 3 | *SLC2A2* | T/A | 86.5 |  | 86.9 |
| **rS3191349** | 7 | *DGKB/TMEM195* | G/T | 45.2 |  | 45.2 |
| **rs4607517** | 7 | *GCK* | G/A | 83.1 |  | 82.6 |
| **rs11558471** | 8 | *SLC30A8* | A/G | 69.1 |  | 67.1 |
| **rs7034200** | 9 | *GLIS3* | A/C | 50.1 |  | 49.1 |
| **rs4506565** | 10 | *TCF7L2* | A/T | 68.7 |  | 69.5 |
| **rs10885122** | 10 | *ADRA2A* | G/T | 87.9 |  | 88.0 |
| **rs174550** | 11 | *FADS1* | T/C | 65.6 |  | 65.6 |
| **rs7944584** | 11 | *MADD* | A/T | 72.1 |  | 73.2 |
| **rs11605924** | 11 | *CRY2* | C/A | 51.3 |  | 53.8 |
| **rs11071657** | 15 | *C2CD4B* | A/G | 62.2 |  | 63.3 |
| **rs35767** | 12 | *IGF1* | A/G | 15.2 |  | 14.6 |

SNP, single-nucleotide polymorphism

Table S3.3. Allele frequencies of 16 glucose metabolism–relevant SNPs, stratified by obesity (measured via waist/hip)

| **SNP** | **Chromosome** | **Nearest gene** | **Allele**  **(effect/baseline)** | **Effect allele frequency** | | |
| --- | --- | --- | --- | --- | --- | --- |
|  |  |  |  | **Non-obese group**  **(w/h ≤ 0.85)** |  | **Obese group**  **(w/h > 0.85)** |
|  |  |  |  | **(n = 3,712)** |  | **(n = 1,667)** |
| **rs340874** | 1 | *PROX1* | C/T | 55.1 |  | 55.8 |
| **rs560887** | 2 | *G6PC2* | T/C | 29.1 |  | 30.6 |
| **rs780094** | 2 | *GCKR* | C/T | 60.1 |  | 59.8 |
| **rs11708067** | 3 | *ADCY5* | A/G | 76.8 |  | 77.2 |
| **rs11920090** | 3 | *SLC2A2* | T/A | 86.8 |  | 86.4 |
| **rS3191349** | 7 | *DGKB/TMEM195* | G/T | 45.3 |  | 44.9 |
| **rs4607517** | 7 | *GCK* | G/A | 82.8 |  | 83.1 |
| **rs11558471** | 8 | *SLC30A8* | A/G | 68.3 |  | 68.1 |
| **rs7034200** | 9 | *GLIS3* | A/C | 50.2 |  | 48.5 |
| **rs4506565** | 10 | *TCF7L2* | A/T | 69.1 |  | 68.9 |
| **rs10885122** | 10 | *ADRA2A* | G/T | 88.2 |  | 87.5 |
| **rs174550** | 11 | *FADS1* | T/C | 65.8 |  | 65.3 |
| **rs7944584** | 11 | *MADD* | A/T | 72.5 |  | 72.7 |
| **rs11605924** | 11 | *CRY2* | C/A | 51.7 |  | 54.0 |
| **rs11071657** | 15 | *C2CD4B* | A/G | 62.5 |  | 63.0 |
| **rs35767** | 12 | *IGF1* | A/G | 14.8 |  | 15.1 |

SNP, single-nucleotide polymorphism; w/h, waist-to-hip ratio

Table S3.4. Allele frequencies of 16 glucose metabolism–relevant SNPs, stratified by physical activity level

| **SNP** | **Chromosome** | **Nearest gene** | **Allele**  **(effect/baseline)** | **Effect allele frequency** | | |
| --- | --- | --- | --- | --- | --- | --- |
|  |  |  |  | **High physical activity group**  **(MET ≥ 10)** |  | **Low physical activity group**  **(MET < 10)** |
|  |  |  |  | **(n = 2,344)** |  | **(n = 3,035)** |
| **rs340874** | 1 | *PROX1* | C/T | 54.5 |  | 56.0 |
| **rs560887** | 2 | *G6PC2* | T/C | 30.1 |  | 29.1 |
| **rs780094** | 2 | *GCKR* | C/T | 59.6 |  | 60.4 |
| **rs11708067** | 3 | *ADCY5* | A/G | 76.6 |  | 77.1 |
| **rs11920090** | 3 | *SLC2A2* | T/A | 87.2 |  | 86.3 |
| **rS3191349** | 7 | *DGKB/TMEM195* | G/T | 45.4 |  | 45.1 |
| **rs4607517** | 7 | *GCK* | G/A | 82.7 |  | 83.1 |
| **rs11558471** | 8 | *SLC30A8* | A/G | 69.2 |  | 67.4 |
| **rs7034200** | 9 | *GLIS3* | A/C | 49.6 |  | 49.7 |
| **rs4506565** | 10 | *TCF7L2* | A/T | 69.0 |  | 69.0 |
| **rs10885122** | 10 | *ADRA2A* | G/T | 87.7 |  | 88.1 |
| **rs174550** | 11 | *FADS1* | T/C | 65.9 |  | 65.4 |
| **rs7944584** | 11 | *MADD* | A/T | 72.7 |  | 72.5 |
| **rs11605924** | 11 | *CRY2* | C/A | 52.7 |  | 52.2 |
| **rs11071657** | 15 | *C2CD4B* | A/G | 63.2 |  | 62.2 |
| **rs35767** | 12 | *IGF1* | A/G | 15.5 |  | 14.5 |

SNP, single-nucleotide polymorphism

Table S3.5. Allele frequencies of 16 glucose metabolism–relevant SNPs, stratified by dietary fat intake

| **SNP** | **Chromosome** | **Nearest gene** | **Allele**  **(effect/baseline)** | **Effect allele frequency** | | |
| --- | --- | --- | --- | --- | --- | --- |
|  |  |  |  | **Low dietary fat intake group**  **(< 40.0% calories from fat)** |  | **High dietary fat intake group**  **(≥ 40.0% calories from fat)** |
|  |  |  |  | **(n = 4,325)** |  | **(n = 1,054)** |
| **rs340874** | 1 | *PROX1* | C/T | 55.3 |  | 55.5 |
| **rs560887** | 2 | *G6PC2* | T/C | 29.6 |  | 29.4 |
| **rs780094** | 2 | *GCKR* | C/T | 59.9 |  | 60.6 |
| **rs11708067** | 3 | *ADCY5* | A/G | 77.2 |  | 75.8 |
| **rs11920090** | 3 | *SLC2A2* | T/A | 87.0 |  | 85.4 |
| **rS3191349** | 7 | *DGKB/TMEM195* | G/T | 45.0 |  | 45.9 |
| **rs4607517** | 7 | *GCK* | G/A | 82.9 |  | 82.7 |
| **rs11558471** | 8 | *SLC30A8* | A/G | 68.2 |  | 68.4 |
| **rs7034200** | 9 | *GLIS3* | A/C | 49.5 |  | 50.3 |
| **rs4506565** | 10 | *TCF7L2* | A/T | 69.3 |  | 67.8 |
| **rs10885122** | 10 | *ADRA2A* | G/T | 88.3 |  | 86.5 |
| **rs174550** | 11 | *FADS1* | T/C | 65.9 |  | 64.3 |
| **rs7944584** | 11 | *MADD* | A/T | 72.8 |  | 71.5 |
| **rs11605924** | 11 | *CRY2* | C/A | 52.7 |  | 51.2 |
| **rs11071657** | 15 | *C2CD4B* | A/G | 62.8 |  | 61.9 |
| **rs35767** | 12 | *IGF1* | A/G | 15.0 |  | 14.6 |

SNP, single-nucleotide polymorphism
