# Supplementary material for: Effect of genetic variants and traits related to glucose metabolism and their interaction with obesity on breast and colorectal cancer risk among postmenopausal women
Source: BMC Cancer. 2017 Apr 26;17:290. doi: 10.1186/s12885-017-3284-7 (PMC5405540; doi:10.1186/s12885-017-3284-7)
Supplement: Supplementary file 4 — Mediation effect of insulin on the relationship between glucose metabolism–relevant SNPs and cancer risk. Table S4.1. Mediation effect of insulin on the relationship between glucose metabolism–relevant SNPs and breast cancer risk, stratified by obesity status and obesity-related factors. Table S4.2. Mediation effect of insulin on the relationship between glucose metabolism–relevant SNPs and CRC risk, stratified by obesity status and obesity-related factors. (DOC 139 kb) [file 12885_2017_3284_MOESM4_ESM.doc]

Table S4.1. Mediation effect of **insulin** on the relationship between **glucose metabolism–relevant SNPs and breast cancer risk**, stratified by obesity status and obesity-related factors

|  |  |  | **Favorable Energy Balance Group** | | | | | | | | | | | | | |  | **Unfavorable Energy Balance Group** | | | | | | | | | | | | | |
| --- | --- | --- | --- | --- | --- | --- | --- | --- | --- | --- | --- | --- | --- | --- | --- | --- | --- | --- | --- | --- | --- | --- | --- | --- | --- | --- | --- | --- | --- | --- | --- |
|  |  |  | **Direct effect** | | | |  | **Indirect effect** | | | |  | **Total effect** | | | |  | **Direct effect** | | | |  | **Indirect effect** | | | |  | **Total effect** | | | |
| **SNP** | **Nearest gene** | **Effect allele/**  **Other allele** | **Breast cancer risk in relation to SNP through pathways other than *insulin*** | | | |  | **Breast cancer risk in relation to SNP through *insulin*** | | | |  | **Breast cancer risk in relation to SNP** | | | |  | **Breast cancer risk in relation to SNP through pathways other than *insulin*** | | | |  | **Breast cancer risk in relation to SNP through *insulin*** | | | |  | **Breast cancer risk in relation to SNP** | | | |
|  |  |  | **HR‡** | **95% CI** | | |  | **Effect size‡** | **95% CI** | | |  | **HR‡** | **95% CI** | | |  | **HR‡** | **95% CI** | | |  | **Effect size‡** | **95% CI** | | |  | **HR‡** | **95% CI** | | |
|  |  |  | **BMI§** | | | | | | | | | | | | | | | | | | | | | | | | | | | | |
| rs560887 | *G6PC2* | T/C | 1.16 | (0.93 | - | 1.45) |  | 0.00 | (-0.002 | **-** | 0.002) |  | 1.12 | (0.90 | - | 1.39) |  | **1.38** | **(1.06** | **-** | **1.78)** |  | -0.004 | (-0.01 | **-** | 0.02) |  | **1.35** | **(1.05** | **-** | **1.74)** |
|  |  |  | **Waist¶** | | | | | | | | | | | | | | | | | | | | | | | | | | | | |
| rs560887 | *G6PC2* | T/C | 1.11 | (0.87 | - | 1.42) |  | -0.01 | (-0.02 | - | 0.01) |  | 1.10 | (0.86 | - | 1.40) |  | **1.39** | **(1.10** | **-** | **1.75)** |  | 0.003 | (-0.01 | - | 0.01) |  | **1.33** | **(1.06** | **-** | **1.66)** |
|  |  |  | **w/h Ratio€** | | | | | | | | | | | | | | | | | | | | | | | | | | | | |
| rs560887 | *G6PC2* | T/C | 1.13 | (0.91 | - | 1.41) |  | 0.004 | (-0.02 | - | 0.01) |  | 1.10 | (0.89 | - | 1.36) |  | **1.46** | **(1.12** | **-** | **1.91)** |  | -0.004 | (-0.002 | - | 0.01) |  | **1.42** | **(1.10** | **-** | **1.85)** |
| rs35767 | *IGF1* | A/G | 0.94 | (0.71 | - | 1.25) |  | 0.002 | (-0.01 | - | 0.004) |  | 0.93 | (0.70 | - | 1.23) |  | **1.47** | **(1.07** | **-** | **2.03)** |  | -0.002 | (-0.004 | - | 0.01) |  | **1.48** | **(1.08** | **-** | **2.03)** |
|  |  |  | **Dietary fat intake†** | | | | | | | | | | | | | | | | | | | | | | | | | | | | |
| rs560887 | *G6PC2* | T/C | 1.17 | (0.97 | - | 1.41) |  | 0.01 | (-0.02 | - | 0.001) |  | 1.14 | (0.95 | - | 1.37) |  | **1.60** | **(1.09** | **-** | **2.33)** |  | 0.002 | (-0.01 | - | 0.01) |  | **1.59** | **(1.10** | **-** | **2.31)** |

BMI, body mass index; CI, confidence interval; HR, hazard ratio; SNP, single–nucleotide polymorphism; w/h ratio, waist-to-hip ratio.

Note: Proportions explained by insulin for SNP–breast cancer risk association for **rs560887** (33.3%,10%, 23.1%, and 21.4% among non-obese group [BMI < 30, waist ≤ 88 cm, w/h ≤ 0.85, and < 40% calories from fat, respectively]; 8.6%, 18.2%, 9.5%, and 1.7% among obese-group [BMI ≥ 30, waist > 88 cm, w/h > 0.85, and ≥ 40% calories from fat, respectively]), and for **rs35767** (1.5% in w/h ≤ 0.85; 1.4% in w/h > 0.85). Only SNPs having statistically significant results are included. Numbers in bold face are statistically significant.

‡ Multivariate regression was adjusted by covariates (age, education, family history of diabetes mellitus, family history of breast cancer, cardiovascular disease ever, hypertension ever, high cholesterol requiring pills ever, total Healthy Eating Index-2005 score, dietary alcohol and total sugars per day, smoking status, lifetime partner, depressive symptom, oral contraceptive use, history of hysterectomy or oophorectomy, age at menarche, age at menopause, pregnancy history, breastfeeding at least one month, and hormone therapy); effect-modifier variables (physical activity, BMI, and w/h ratio), when not evaluated as effect modifier variables, were adjusted as a covariate; when stratified via waist circumference, w/h ratio was not adjusted.

§ Participants stratified by BMI as non-obese (BMI < 30, n = 3,675) or obese (BMI ≥ 30, n = 1,704); interaction test presented for the effect of BMI on the association between breast cancer and rs560887 (effect size -0.38, p-value 0.28).

¶ Participants stratified by waist circumference as non-obese (waist ≤ 88 cm; n = 3,042) or obese (waist > 88 cm; n = 2,337); interaction test presented for the effect of waist circumference on the association between breast cancer and rs560887 (effect size -0.55, p-value 0.13).

€ Participants stratified by w/h as non-obese (w/h ≤ 0.85; n = 3,712) or obese (w/h > 0.85; n = 1,667); interaction tests presented for the effect of w/h on the association between breast cancer and rs560887 (effect size -0.57, p-value 0.11) and rs35767 (effect size 0.54, p-value 0.01).

† Participants stratified by dietary fat intake as non-obese (< 40% calories from fat; n = 4,325) or obese (≥ 40% calories from fat; n = 1,054); interaction test presented for the effect of dietary fat intake on the association between breast cancer and rs560887 (effect size -0.45, p-value 0.26).

Table S4.2. Mediation effect of **insulin** on the relationship between **glucose metabolism–relevant SNPs and CRC risk**, stratified by obesity status and obesity-related factors

|  |  |  | **Favorable Energy Balance Group** | | | | | | | | | | | | | |  | **Unfavorable Energy Balance Group** | | | | | | | | | | | | | |
| --- | --- | --- | --- | --- | --- | --- | --- | --- | --- | --- | --- | --- | --- | --- | --- | --- | --- | --- | --- | --- | --- | --- | --- | --- | --- | --- | --- | --- | --- | --- | --- |
|  |  |  | **Direct effect** | | | |  | **Indirect effect** | | | |  | **Total effect** | | | |  | **Direct effect** | | | |  | **Indirect effect** | | | |  | **Total effect** | | | |
| **SNP** | **Nearest gene** | **Effect allele/**  **Other allele** | **CRC risk in relation to SNP through pathways other than *insulin*** | | | |  | **CRC risk in relation to SNP through *insulin*** | | | |  | **CRC risk in relation to SNP** | | | |  | **CRC risk in relation to SNP through pathways other than *insulin*** | | | |  | **CRC risk in relation to SNP through *insulin*** | | | |  | **CRC risk in relation to SNP** | | | |
|  |  |  | **HR‡** | **95% CI** | | |  | **Effect size‡** | **95% CI** | | |  | **HR‡** | **95% CI** | | |  | **HR‡** | **95% CI** | | |  | **Effect size‡** | **95% CI** | | |  | **HR‡** | **95% CI** | | |
|  |  |  | **BMI§** | | | | | | | | | | | | | | | | | | | | | | | | | | | | |
| rs4607517 | *GCK* | G/A | **0.79** | **(0.63** | **-** | **0.98)** |  | 0.001 | (-0.004 | **-** | 0.01) |  | **0.80** | **(0.64** | **-** | **1.00)** |  | 1.08 | (0.75 | - | 1.54) |  | -0.004 | (-0.02 | **-** | 0.01) |  | 1.06 | (0.75 | - | 1.51) |
| rs174550 | *FADS1* | T/C | 1.07 | (0.89 | - | 1.29) |  | <0.001 | (-0.002 | **-** | 0.002) |  | 1.05 | (0.87 | - | 1.27) |  | **1.34** | **(1.00** | **-** | **1.80)** |  | 0.01 | (-0.01 | **-** | 0.03) |  | **1.37** | **(1.02** | **-** | **1.83)** |
| rs11605924 | *CRY2* | C/A | **0.81** | **(0.68** | **-** | **0.97)** |  | -0.001 | (-0.01 | **-** | 0.01) |  | **0.82** | **(0.69** | **-** | **0.98)** |  | 1.08 | (0.82 | - | 1.41) |  | -0.003 | (-0.01 | **-** | 0.02) |  | 1.07 | (0.82 | - | 1.39) |
|  |  |  | **Waist¶** | | | | | | | | | | | | | | | | | | | | | | | | | | | | |
| rs340874 | *PROX1* | C/T | **0.81** | **(0.66** | **-** | **0.99)** |  | 0.002 | (-0.01 | **-** | 0.01) |  | 0.82 | (0.67 | - | 1.00) |  | 1.21 | (0.98 | - | 1.50) |  | <0.001 | (-0.004 | - | 0.003) |  | 1.20 | (0.97 | - | 1.49) |
| rs2191349 | *DGKB/TMEM195* | G/T | 0.98 | (0.80 | - | 1.21) |  | <0.001 | (-0.01 | **-** | 0.01) |  | 1.00 | (0.81 | - | 1.23) |  | **1.23** | **(1.00** | **-** | **1.52)** |  | 0.001 | (-0.01 | **-** | 0.01) |  | 1.20 | (0.97 | - | 1.48) |
| rs174550 | *FADS1* | T/C | 1.07 | (0.86 | - | 1.33) |  | -0.001 | (-0.01 | **-** | 0.01) |  | 1.03 | (0.83 | - | 1.28) |  | 1.24 | (0.99 | - | 1.56) |  | 0.002 | (-0.01 | - | 0.02) |  | **1.25** | **(1.00** | **-** | **1.57)** |
| rs11605924 | *CRY2* | C/A | **0.80** | **(0.66** | **-** | **0.99)** |  | 0.002 | (-0.01 | **-** | 0.01) |  | **0.81** | **(0.66** | **-** | **0.99)** |  | 0.95 | (0.77 | - | 1.18) |  | -0.002 | (-0.01 | **-** | 0.01) |  | 0.97 | (0.79 | - | 1.20) |
|  |  |  | **waist/hip Ratio€** | | | | | | | | | | | | | | | | | | | | | | | | | | | | |
| rs10885122 | *ADRA2A* | G/T | 0.82 | (0.63 | - | 1.07) |  | 0.002 | (-0.01 | - | 0.01) |  | 0.84 | (0.64 | - | 1.08) |  | 1.56 | (0.99 | - | 2.44) |  | -0.001 | (-0.01 | - | 0.004) |  | **1.58** | **(1.01** | **-** | **2.48)** |
|  |  |  | **Physical activity level¥** | | | | | | | | | | | | | | | | | | | | | | | | | | | | |
| rs4607517 | *GCK* | G/A | **0.71** | **(0.54** | **-** | **0.94)** |  | -0.001 | (-0.01 | - | 0.01) |  | **0.72** | **(0.55** | **-** | **0.95)** |  | 0.98 | (0.76 | - | 1.27) |  | 0.003 | (-0.01 | - | 0.01) |  | 0.99 | (0.76 | - | 1.28) |
|  |  |  | **Dietary fat intake†** | | | | | | | | | | | | | | | | | | | | | | | | | | | | |
| rs4607517 | *GCK* | G/A | 0.94 | (0.76 | - | 1.16) |  | <0.001 | (-0.01 | - | 0.01) |  | 0.94 | (0.76 | - | 1.17) |  | **0.65** | **(0.43** | **-** | **0.99)** |  | <0.001 | (-0.003 | - | 0.003) |  | **0.66** | **(0.43** | **-** | **1.00)** |
| rs11558471 | *SLC30A8* | A/G | 0.96 | (0.81 | - | 1.15) |  | -0.001 | (-0.01 | - | 0.004) |  | 0.95 | (0.80 | - | 1.12) |  | **1.57** | **(1.05** | **-** | **2.34)** |  | 0.001 | (-0.01 | - | 0.01) |  | **1.60** | **(1.07** | **-** | **2.40)** |

BMI, body mass index; CI, confidence interval; CRC, colorectal cancer; HR, hazard ratio; SNP, single–nucleotide polymorphism; w/h ratio, waist-to-hip ratio.

Note: Proportions explained by insulin for SNP–CRC risk association for **rs4607517** (1.4%, 1.2%, and 0.5% among non-obese group [BMI < 30, MET ≥ 10, and < 40% calories from fat, respectively]; 23.0%, 0.8%, and 0.6% among obese-group [BMI ≥ 30, MET > 10, and ≥ 40% calories from fat, respectively]), for **rs174550** (34.6% and N/A[> 100%] among non-obese group [BMI < 30 and waist ≤ 88 cm, respectively]; 5.9% and 4.0% among obese-group [BMI ≥ 30 and waist > 88 cm, respectively]), for **rs11605924** (1.2% and 1.2% among non-obese group [BMI < 30 and waist ≤ 88 cm, respectively]; 14.3% and 2.1% among obese-group [BMI ≥ 30 and waist > 88 cm, respectively]), for **rs340874** (1.2% in waist ≤ 88 cm; 5.0% in waist > 88 cm), for **rs2191349** (1.0% in waist ≤ 88 cm; 15% in waist > 88 cm), for **rs10885122** (1.2% in w/h ≤ 0.85; 4.6% in w/h > 0.85), and for **rs11558471** (1.7% in < 40% calories from fat; 5.5% in ≥ 40% calories from fat). Only SNPs having statistically significant results are included. Numbers in bold face are statistically significant.

‡ Multivariate regression was adjusted by covariates (age, education, family history of diabetes mellitus, family history of colorectal cancer, cardiovascular disease ever, hypertension ever, high cholesterol requiring pills ever, total Healthy Eating Index-2005 score, dietary alcohol and total sugars per day, smoking status, lifetime partner, depressive symptom, oral contraceptive use, history of hysterectomy or oophorectomy, age at menarche, age at menopause, pregnancy history, breastfeeding at least one month, and hormone therapy); effect-modifier variables (physical activity, BMI, and w/h ratio), when not evaluated as effect modifier variables, were adjusted as a covariate; when stratified via waist circumference, w/h ratio was not adjusted.

§ Participants stratified by BMI as non-obese (BMI < 30, n = 3,675) or obese (BMI ≥ 30, n = 1,704); interaction tests presented for the effect of BMI on the association between CRC and rs4607517 (effect size -0.30, p-value 0.65), rs174550 (effect size -0.70, p-value 0.09), and rs11605924 (effect size -0.38, p-value 0.15).

¶ Participants stratified by waist circumference as non-obese (waist ≤ 88 cm; n = 3,042) or obese (waist > 88 cm; n = 2,337); interaction tests presented for the effect of waist circumference on the association between CRC and rs340874 (effect size 0.49, p-value 0.04), rs2191349 (effect size -0.21, p-value 0.38), rs174550 (effect size -0.89, p-value 0.01), and rs11605924 (effect size -0.42, p-value 0.09).

€ Participants stratified by w/h as non-obese (w/h ≤ 0.85; n = 3,712) or obese (w/h > 0.85; n = 1,667); interaction test presented for the effect of w/h on the association between CRC and rs10885122 (effect size 0.59, p-value 0.02).

¥ Participants stratified by physical activity level as non-obese (MET ≥ 10; n = 2,344) or obese (MET < 10; n = 3,035); interaction test presented for the effect of physical activity on the association between CRC and rs4607517 (effect size 0.94, p-value 0.14).

† Participants stratified by dietary fat intake as non-obese (< 40% calories from fat; n = 4,325) or obese (≥ 40% calories from fat; n = 1,054); interaction tests presented for the effect of dietary fat intake on the association between CRC and rs4607517 (effect size 1.45, p-value 0.01) and rs11558471 (effect size -1.53, p-value 0.04).
